# High-speed threat detection in 5G SDN with particle swarm optimizer integrated GRU-driven generative adversarial network

**DOI:** 10.1038/s41598-025-95011-z

**Published:** 2025-03-23

**Authors:** R. Shameli, Sujatha Rajkumar

**Affiliations:** School of Electronics Engineering, Vellore Institute of Technology, Vellore, India

**Keywords:** 5G, Deep learning, Gated recurrent unit, Generative adversarial network, Intrusion detection, Particle swarm optimization, Software defined network, Engineering, Mathematics and computing

## Abstract

Detecting attacks in 5G software-defined network (SDN) environments requires a comprehensive approach that leverages traditional security measures, such as firewalls, intrusion prevention systems, and specialized techniques personalized to the unique characteristics of a 5G network. The attack detection in 5G SDN involves Machine learning (ML) and Deep learning (DL) algorithms to analyze large volumes of network data and identify patterns indicative of attacks. The study’s main objective is to develop an efficient DL model to improve the detection performance and respond to security breaches effectively in a 5G SDN environment. The DL model integrates the Particle Swarm Optimizer-Gated Recurrent Unit Layer-Generative Adversarial Network-Intrusion Detection System classifier (PSO-GRUGAN-IDS). The PSO optimizes the network weight of the GAN model to improve the backpropagation while generating the synthetic data (attack data) in the generator model using GRU. The discriminator model uses the PSO-optimized generator model to produce synthetic and real attack data to forecast the attack. Finally, a deep classification (IDS) model is trained using a GRU network with a GAN model-produced attack data and real data to classify whether the SDN traffic is malicious or normal. Moreover, the performance of this model is evaluated using the InSDN dataset and compared with existing DL model-based intrusion detection approaches and the results demonstrate a significantly higher accuracy rate of 98.4%, precision rate of 98%, recall rate of 98.5%, less detection time of 2.464 s, lesser Log loss rate of 1.0 and more metrics instilling confidence in the effectiveness of the proposed method.

## Introduction

**Fig. 1:**
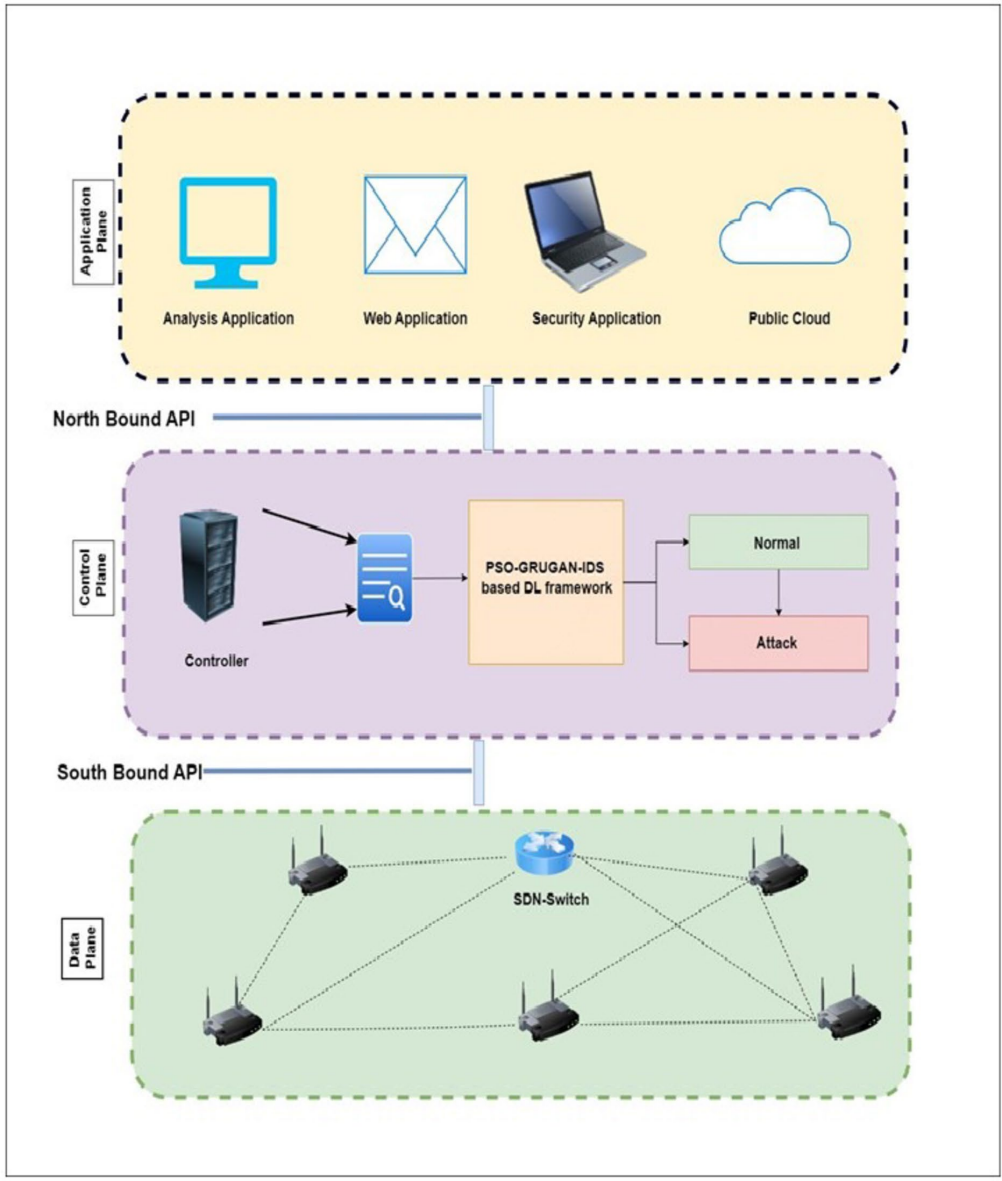
PSO-GRUGAN-IDS model of intrusion detection framework placement in 5G SDN network.

Integrating Intrusion Detection Systems (IDS) in 5G software-defined networks (SDN) is necessary for maintaining robust network security and improving the quality of service (QoS) in networks^1^. 5G networks may be made secure and resilient by using an intrusion detection system (IDS) that uses SDN’s programmable and dynamic features to enable real-time threat detection. This paper addresses these challenges and implements best practices for effective IDS deployment in this advanced network environment. The architecture of 5G networks leveraging SDN is typically divided into three primary layers, each designed to perform different network functions, providing a clear structure for implementing and managing the network effectively. By understanding and addressing the security needs at each layer of the 5G SDN network in Fig. 1, organizations can create a robust intrusion detection (ID) and response strategy that enhances overall network security and resilience. A network monitoring application in the application layer detects an unusual traffic pattern indicative of a potential Distributed Denial of Service (DDoS) attack^2,3^. The control layer monitoring applications (Ex, IDS tools) send alerts and decide to reroute the traffic to mitigate the attack. The infrastructure layer’s SDN controller^4–7^ uses the southbound APIs to update the switch and routers, flow rules, redirecting malicious traffic to a honeypot for further analysis while ensuring that traffic is unaffected. The interaction between these layers was determined using northbound and southbound APIs. Northbound APIs enable communication between the application and the control layers. Applications use these APIs to request network resources and send instructions to an SDN controller. Southbound APIs allow communication between the control layer and the infrastructure layer.

The SDN controller uses these APIs to configure network devices and manage traffic flows. ID in the control plane of a 5G network using SDN^8^ is crucial for ensuring the security and integrity of the network. The control plan in SDN is responsible for making decisions about how packets should flow through the network; any compromise here can lead to significant security breaches. The controller comprehensively views the entire network, enabling efficient traffic management and security monitoring. The integration IDS with SDN controller plane to monitor control plane traffic. Use a controller’s APIs to gather data on flow entries, network configurations, and traffic patterns. The main characteristics of the 5G network are high bandwidth and low latency, network slicing, and edge computing. Latency reduction and improving response time are the challenges in the 5G network. This system has high device density, and diverse applications increase the attack surface. The low latency requirement of a 5G network necessitates a real-time detection system and response to threats. The centralized nature of the SDN control plane makes it a high-value target for attacks. Compromise of the SDN controller^9^ can lead to widespread network disruption. These limitations in the 5G SDN networks require centralized ID^10^, distributed ID^11^, ML^12^, DL^13^, artificial intelligence (AI) tools^14^, and network slicing security. Centralized ID systems require controller-based detection systems. It embeds IDS functionalities within the SDN controller to leverage its global view and centralized control. The flow monitoring mechanism monitors and analyzes flow entries in the SDN controller to detect abnormal patterns indicative of malicious activities. The distributed detection system deploys edge-based IDS at the network edge to monitor traffic closer to the source, providing early detection and reducing the load on the central controller.

Collaborative detection uses a network of distributed IDS nodes that communicate and collaborate to detect and respond to threats. So, adequate DL^15^, ML^16^, and AI models^17^ need to be developed for anomaly detection. It utilizes ML/DL models to establish a baseline of normal behavior and detect deviations. Predictive analysis approaches implement AI techniques to predict potential attacks based on historical data and patterns. This approach uses statistical, ML, and DL techniques^18,19^ to detect unknown threats by identifying deviations from normal behavior.

Moreover, the analysis helps the response system to use SDN capabilities to reroute or isolate suspicious traffic quickly—the automatic response systems of SDN control to respond to certain types of IDS-detected threats^20^. Snort, Zeek, and bro are the popular IDS tools^21^ in the market that can serve as IDS in the SDN network. However, these tools require colossal investment to integrate and implement in real-time. So, this research focuses on developing a cost-effective IDS tool to continuously monitor network traffic and adjust detection strategies based on emerging threats and network changes. Tensorflow and Python libraries are the prevalent source frameworks for implementing various ID algorithms. Benign users are legitimate network users who engage in normal, non-malicious activities (attacks). Distributed Denial of Service (DDoS) attacks^22^, User root (U2R) attacks^23^, and Brute force attacks (BFA) pose significant attacks due to the crucial functionalities provided by the 5G SDN network. These three attacks are common attacks occurring in SDN layers. The DoS, DDoS, Malware, and Probe attacks impact all the aspects of SDN (Externally except DDoS), including the data plane (DP), southbound interface (SI), controller, northbound interface (NI), and application plane (AP). Web attacks create impacts on NI and AP. A brute force attack impacts all the elements except SI, and the exploitation attack impacts the controller, NI, and AP. DDoS attacks aim to overwhelm network resources, services, or infrastructure with massive traffic, rendering them unavailable to legitimate (Benign) users. The BFAs^24^ involve attackers trying numerous combinations of usernames and passwords to gain unauthorized access to systems or services. The U2R attacks^25^ include attackers gaining unauthorized root or administrative access to a system, usually by exploiting application or operating system vulnerabilities. Distinguishing between benign and malicious behavior is crucial for the effective functioning of an IDS^26^. Understanding benign user behavior helps reduce false positive rates, where legitimate actions are mistakenly flagged as threats, and enhances the IDS model’s overall accuracy. Effectively managing benign users in an IDS involves establishing accurate baselines, implementing context-aware detection mechanisms, and continuously refining detection methods to reduce false positives. By understanding and monitoring benign user behaviors, IDS can more accurately identify potential threats while minimizing disruptions to legitimate activities, ensuring a secure and efficient network environment.

This study developed a DL-based hybrid IDS model, and the InSDN dataset is used to evaluate the DL model on an SDN network’s traffic abnormality detection problem. Additionally, the proposed model is assessed using two other datasets, EDGE_IIoT and BoT-IoT, to achieve improved performance accuracy. The SDN controller plane trains the DL model with internal and external attacks. The internal attacks come from the organization’s internal users (who have full access to their network), and the external attacks come from outside the network. While most research focuses on SDN network-based IDS, the increasing use of Internet of Things (IoT) devices and cloud-based applications presents a growing challenge for network security providers.

The Motivation and Contributions of the Work are,

- The critical challenge for 5G SDN network developers is to create efficient intrusion detection, continuous network traffic monitoring, and immediate response to security breaches.
- As SDN plays a vital role in 5G it is essential to safeguard and Preserve the SDN infrastructure which guarantees peak performance and avoids problems such as bottlenecks and congestion.
- SDN security contributes to protecting the data against breaches and illegal access.
- This study develops an efficient intrusion system to safeguard the 5G SDN network from security breaches.
- In this context, the study introduces a novel DL-based IDS framework, the PSO-GRUGAN-IDS model, which significantly enhances attack detection accuracy and reduces the false positive rate.
- The model’s generated synthetic traffic samples strengthen the SDN security mechanism with access control by optimizing the network performance.
- The DL model-based attack detection mechanism is designed to improve the security mechanism in the SDN controller plane to enable centralized monitoring, analysis, and enforcement of security policies across the entire 5G SDN infrastructure.

The following are the primary objectives of this paper.

- The DL model has recently played a key role in attack detection due to their advanced capabilities in handling sophisticated attacks.
- The DL model can reduce false positive and negative rates and achieve higher Performance metrics of accuracy, precision, Recall, F1-score, and Throughput.
- It can improve detection and reduce the burden on security teams.
- The DL model can scale with the growth of the network infrastructure, maintaining effectiveness as the volume and complexity of the traffic increase.
- Robust DL model solutions address the boundaries of traditional detection methods, safeguarding a more secure and strong SDN network environment.
- DL models can mechanically learn the extracted features from traffic data, simplifying detection.
- This automation can improve the detection system’s efficiency, allowing it to adapt quickly to new threats without extensive reconfiguration.

The remainder of the section is arranged as follows: Part 2 discusses the literature review on different IDS and their methodologies. Part 3 describes the various functionalities of the PSO-GRUGAN-IDS model, and part 4 discusses the model performance evaluation results. Part 5 discusses the research findings and future directions as a final point.

## Literature review

Shahid Allah Bakhsh et al.^27^ prepared a performance analysis of Feedforward neural network (FFNN), Long short-term memory (LSTM), and random, artificial neural network (RandNN) models for cyber threats detection in the IoT environment. The performance analysis demonstrated that the FFNN model outperformed the other two DL models in managing the complexity of IoT data. D. Javeed et al.^28^ presented a novel SDN-based IDS using a DL model to separate the control and data planes for a smart consumer electronics network (SCEN). The DL model utilizes the Cuda-enabled bidirectional LSTM (Cu-BLSTM). It is designed to identify different attack types in the SCEN. The simulation analysis demonstrated that the DL model-based framework provides solutions for recent security issues in the network.

Maddu M et al.^29^ utilized the DL-based IDS for SDN networks. The IDS approach uses CenterNet for feature extraction. Deep convolutional generative adversarial network (DCGAN) performs data augmentation to reduce class imbalance issues. Slim mould algorithm (SMA) optimized ResNet152V2 is developed to classify the attacks in InSDN and Edge IoT datasets. Once the attack is detected, a predefined defense module restores the connectivity of the SDN network. Khekare Ganesh et al.^30^ integrate the GAN with a Recurrent neural network (GAN-RNN) to manage traffic engineering and accessibility control in the SDN environment. The GAN-RNN’s performance analysis shows the model performs well in flexible rule access management.

D.M. Brandao Lent et al.^31^ propose an anomaly detection system for DDoS attacks using GAN with GRU. This approach designs a mitigation algorithm to stop DDoS attacks from harming the SDN network. This model is evaluated with two datasets, including Orion and CIC DDoS2019. Moreover, the detection performance is evaluated using GRU, LSTM, convolutional, and temporal convolution. Alzughaibi Saud et al.^32^ developed two DL model for IDS. The first model uses a multi-layer perceptron (MLP), and the second uses a PSO-optimized MLP for binary and multiclass attack class classification. These models use the CSE-CIC-IDS2018 dataset to evaluate their efficiency. The analysis shows that these models give better performance for binary classification.

Sundaram K et al.^33^ developed a novel IDS approach to improve IoT security against cyber-attacks in wireless networks. The IDS model integrates the Ant Lion optimization (ALO) with GRU. This approach gives promising results while validating with NSL-KDD and UN-NB15 datasets. Sontakke P V et al.^34^ developed a weight-optimized DNN model to detect and mitigate intrusions. This approach uses two phases: the first phase performs traffic feature extraction and vehicle position extraction. The intrusion detection and mitigation system uses the improved PSO algorithm to increase the DNN model’s network weight. This framework uses a BAIT-based mitigation process. The model performance is compared with five existing DNN models. P F de Araujo-Filho et al.^35^ Prepare an investigation of the performance of GANs for IDS. The GAN-based IDS detects attacks using a temporal convolution network and self-attention mechanism. The IDS leverages edge computing and servers, bringing computation resources closer to end notes.

Vikash Kumar et al.^36^ designed a DL model to deal with imbalanced data and improve attack detection accuracy. This approach combines the Wasserstein conditional GAN (WCGAN) with the XGBoost classifier. The WCGAN model’s balanced data and XGBoost’s gradient penalty help for stable learning. The results show that the Wasserstein variant GAN model achieves a lower loss rate for NSL-KDD, UNSW-NB15, and BoT-IOT datasets. Banitalebi Dehkordi, B. et al.^37^, A novel computer architecture known as SDN is described in this study. As a result, the security of these networks is at risk from various types of threats. DDoS attacks are one of the most recent and severe computer network threats. A statistical & ML is used to create an attack detector. This approach uses entropy to identify attacks based on destination IP, normal distribution, and the dynamic threshold is possible. Hassan A. Alamri et al.^38^ offer a DDoS mitigation strategy to ensure precise attack detection and optimal network resource usage for SDN. This approach integrates the bandwidth management mechanism with the XGBoost classifier. When the threshold is exceeded, the XGBoost algorithm kicks in based on an adjustable frequency profile threshold and bandwidth control algorithm. If the network traffic flow exceeds a threshold, the XGBoost algorithm categorizes it as normal or abnormal. Various data sets were used to evaluate the approach’s effectiveness. M S Elsayed et al.^39^ developed an attack-specific SDN dataset (InSDN) to evaluate IDS performance. The dataset contains several attacks and normal traffic samples and evaluates the performance of different ML models.

Sokkalingam S et al.^40^ introduced a hybrid machine learning (ML) intrusion detection system (IDS) using a 10-fold cross-validation technique for feature selection, reducing the dimensions of the NSL-KDD dataset. The model’s performance was validated with a confusion matrix, and SVM parameters were optimized using a hybrid Harris Hawks Optimization (HHO) and Particle Swarm Optimization (PSO). The optimized SVM model showed superior DDoS detection capabilities. Sumathi S et al.^41^ employed an LSTM network with an autoencoder-decoder deep learning approach, optimized using a hybrid HHO-PSO algorithm. This model outperformed existing literature in attribute selection and performance metrics. Further, Sumathi S et al.^42^ found that integrating features selected by the C4.5 algorithm with SVM and KNN models improved performance. The hybrid C4.5-SVM model achieved an accuracy of 0.9604, surpassing other models. In 2024, Sumathi S et al.^43^ proposed an ANN-based hybrid IDS combining GWO, BPN, and SOM for cloud computing. Using the UNSW-NS 15 dataset, the model achieved a detection accuracy of 99.40%, with minimal false alarms and fast prediction times.

Finally, Sumathi S et al.^44^ addressed stochastic model parameters in ANN-based IDS by introducing the HHOPSO algorithm, improving BPN and MLP IDS models’ accuracy to 97.08% and 97.74% respectively, with high F1 scores. Han et al.^45^ propose a new feature selection (FS) approach, BPSO-SA, combining Binary Particle Swarm Optimization (BPSO) and Simulated Annealing (SA) with Gray Wolf Optimization (GWO) to enhance the LightGBM model for detecting reflective Distributed Denial of Service (DDoS) attacks. BPSO-SA improves global search capabilities, while GWO optimizes LightGBM hyperparameters. Experimental results show the model surpasses conventional methods in accuracy, precision, recall, F1 score, and prediction time. (A) A. E. (B) Donkol et al.^46^ introduce an enhanced long-short-term memory (ELSTM) technique with recurrent neural network (RNN) to address issues like gradient vanishing and overfitting in intrusion detection systems (IDS). Using likely point particle swarm optimization (LPPSO) and ELSTM, the system was validated on datasets like NSL-KDD and UNSW-NB15. Results indicate reduced training time and superior performance compared to LPBoost and deep neural networks (DNNs). Wahab et al.^47^ present a cognitive hybrid-deep learning model for intrusion detection in IoT, leveraging software-defined networking (SDN). The model, trained on N-BaIoT and CICDDoS2019 datasets, demonstrates high accuracy with minimal false positives and efficiently handles IoT resource constraints. The proposed model outperforms the other hybrid-DL models like Cu-GRU + LSTM.

Liu et al.^48^ designed an Adaptive Load Balancing based on Traffic Prediction (ALB-TP) using a GRU-attention model to improve congestion prediction and network scalability. ALB-TP reduces Flow Completion Time (FCT) and increases throughput, showing a 28.2% improvement in prediction accuracy over existing models. Rani et al.^49^ introduce a hybrid deep learning model, DINet, combining a deep temporal convolution network and gated recurrent unit, optimized using the Improved Chimp Optimization Algorithm (IChOA). This model effectively detects intrusions with 97% accuracy and precision, outperforming prior methods in malware detection. Hnamte et al.^50^ propose a deep neural network (DNN) for DDoS detection in SDN environments. The model analyzes network traffic to detect DDoS patterns, showing superior performance over traditional methods with high detection accuracy and low loss rates on datasets like InSDN and CICIDS2018.

Maddu et al.^51^ developed an intrusion detection system using deep learning and DCGAN for data augmentation. The system employs a ResNet152V2 and Slime Mold Algorithm (SMA) to detect network intrusions effectively in InSDN and Edge IIoT datasets, demonstrating robust detection and mitigation capabilities. Aslam et al.^52^ provide a taxonomy of DDoS defense solutions, reviewing 132 ML- and DL-based studies. They highlight the importance of feature selection algorithms and SDN-specific datasets for improving DDoS detection, outlining future research challenges in SDN security.

Most IDS models discussed in this section use highly imbalanced data samples. Most dataset studies use outdated attack datasets, including KDD’99, NSL-KDD, CICIDS-2018, and CSE-CIC-IDS2018. Resulting in a biased ID model towards the majority classes. However, these approaches have yet to impact the detection accuracy improvement. So, this study uses the current SDN network attacks-based dataset while building an IDS model. Most of the SDN attack dataset has class imbalance issues. So, this study utilizes the GAN model to generate synthetic data to avoid imbalance issues. The attack dataset’s temporal nature needs models suitable for efficiently handling the time-dependent attack data. So, this study integrates the GRU layer in GAN to handle the temporal attack data while detecting abnormal traffic behaviors in the SDN system.

The literature review discusses various GAN and deep learning-based IDS models for SDN and IoT environments, focusing on 5G SDN security:

- Model Architectures and Features: Shahid Allah Bakhsh et al.^27^ highlight FFNN’s efficacy for IoT data, though it lacks SDN specificity. D. Javeed et al.^28^ use Cu-BLSTM for SCEN, offering scalability for 5G. Maddu M et al.^29^ and Khekare Ganesh et al.^30^ focus on DCGAN for data augmentation and GAN-RNN for traffic management, respectively. D.M. Brandao Lent et al.^31^ and P F de Araujo-Filho et al.^35^ use GAN with GRU and temporal convolution for DDoS detection and edge computing.
- Handling Imbalanced Data: Maddu M et al.^29^ and Vikash Kumar et al.^36^ use GAN variants to address class imbalance, improving detection accuracy in 5G networks.
- Performance and Optimization: Alzughaibi Saud et al.^32^ and Sundaram K et al.^33^ employ PSO and ALO for optimization, enhancing IDS performance. Hassan A. Alamri et al.^38^ use XGBoost for efficient bandwidth management and attack detection.
- Evaluation of Diverse Datasets: While many studies rely on older datasets, M S Elsayed et al.^39^ and Wahab et al.^47^ utilize newer ones like InSDN, better reflecting current 5G network threats.

The proposed PSO-GRUGAN-IDS model addresses the limitations of previous models by integrating a GRU layer to handle the temporal nature of attack data in SDN and employing PSO for optimal weight initialization. This model aims to enhance detection accuracy and reduce processing time, crucial for the dynamic and high-throughput demands of 5G SDN security. The advancements in GAN-based IDS models have significantly improved the capability to detect and mitigate cyber threats in SDN environments. However, challenges remain in handling imbalanced datasets, real-time processing, and adapting to the evolving 5G landscape. The integration of techniques like PSO and GRU in GAN models, as proposed in the PSO-GRUGAN-IDS model, presents a promising approach to addressing these challenges, enhancing both the accuracy and efficiency of IDS in 5G SDN networks.

## Proposed framework of PSO optimized GRU integrated GAN-IDS model for traffic attack classification approach

Using IDS data for attack detection involves analyzing the logs and alerts generated by the IDS to identify signs of malicious activities. This process involves data collection, preprocessing, analyzing data to detect patterns indicative of attacks, and model evaluation.

**Fig. 2:**
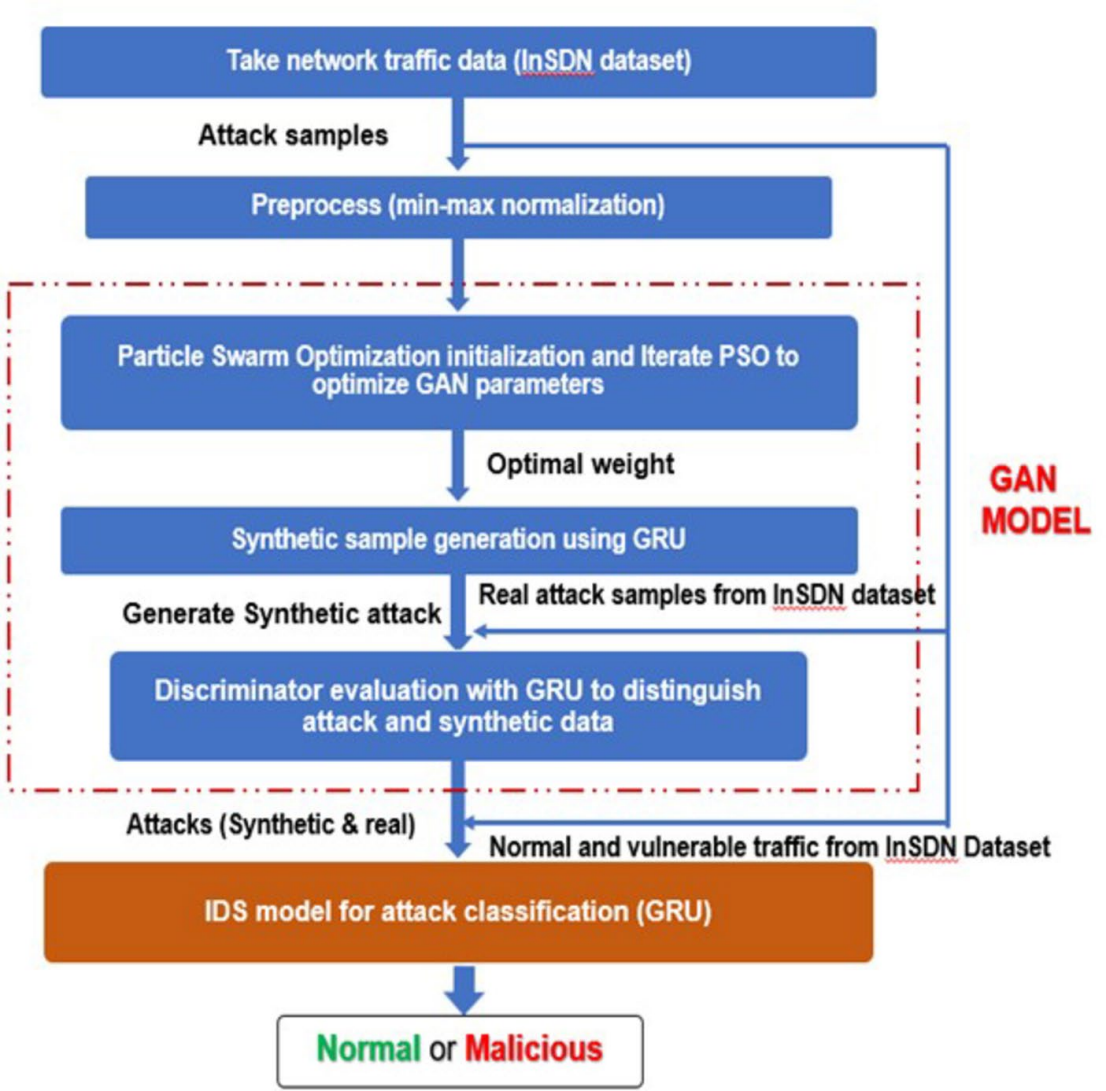
Workflow of PSO-GRUGAN-IDS proposed model for identifying traffic anomalies.

Figure 2 illustrates the dynamic process of the PSO-GRUGAN-IDS model of traffic abnormality detection approach. This approach performs three critical stages: min-max and standard scalar-based normalization, PSO optimized GRU integrated GAN model for attack detection. Finally, the GRUGAN - IDS model detects the real attack traffic and benign traffic. The functionalities of the methodologies used in this approach are given in this section.

### Dataset analysis

This study utilized three different datasets such as InSDN dataset, EDGE_IIoT, and BoT-IoT. The InSDN dataset^39,53^ comprises 68,424 normal and 275,515 attack traffic samples, categorized into normal, metasploitable-2, and OVS groups. The dataset includes 80 features which are categorized into 56 feature clusters.

The Edge-IIoTset dataset^54^, designed for IoT and IIoT cybersecurity, supports centralized and federated learning modes for machine learning-based intrusion detection systems. It spans seven layers: Cloud Computing, Network Functions Virtualization, Blockchain Networks, Fog Computing, Software-Defined Networking, Edge Computing, and IoT/IIoT Perception. Each layer integrates emerging technologies like the ThingsBoard IoT platform, OPNFV, Hyperledger Sawtooth, and ONOS SDN controller. Data is generated from over 10 IoT device types, including temperature sensors, pH meters, and heart rate sensors. The dataset covers 14 attack types across five threat categories: DoS/DDoS, information gathering, man-in-the-middle, injection, and malware attacks, with exploratory analysis provided for machine learning evaluation.

The BoT-IoT dataset^55^, created at UNSW Canberra’s Cyber Range Lab, features normal and botnet traffic with a focus on attacks like DDoS, DoS, keylogging, and data exfiltration. Available in pcap, argus, and CSV formats, it contains over 72 million records, with a 5% subset extracted for ease of handling, totaling about 1.07 GB and 3 million records. This dataset supports detailed labeling and analysis of various attack categories.

For evaluation, 60,000 instances were used for binary classification in the PSO-GRUGAN-IDS model, with 80% for training and 20% for testing. The generator model produced synthetic attack data, combined with real attack and benign data from the InSDN dataset, to assess the model’s ability to distinguish between attack and benign traffic in 5G SDN networks. Table 1. demonstrates only a few sample feature values of normal traffic, U2R, BFA, DDoS, and Probe class attacks.

**Table 1:** Sample of few feature values of normal traffic, U2R, BFA, DDoS, probe class attacks of InSDN dataset.

Flow ID	Src IP	Src Port	Dst IP	Dst Port	Protocol	Timestamp	Flow Duration	Tot Fwd Pkts	Class
185.127.17.56-192.168.20.133-443-53648-6	185.127.17.56	443	192.168.20.133	53,648	6	5/2/2020 13:58	245,230	44	Normal
192.168.3.130-200.175.2.130-38694-4444	192.168.3.130	38,694	200.175.2.130	4444	6	10/1/2020 5:02	269,709	4	U2R
192.168.3.130-200.175.2.130-3632-33747-6	200.175.2.130	33,747	192.168.3.130	3632	6	10/1/2020 5:02	22,194	5	BFA
192.168.3.130–6.234.132.122-0-0-0	6.234.132.122	0	192.168.3.130	0	0	10/1/2020 5:56	22	0	DDoS
192.168.3.130-200.175.2.130-7134-43853-6	200.175.2.130	43,853	192.168.3.130	7134	6	9/1/2020 17:32	5	0	Probe
192.168.3.130-200.175.2.130-41967-4444-6	192.168.3.130	41,967	200.175.2.130	4444	6	10/1/2020 4:41	270,361	6	U2R

### Data preprocessing

Min-max normalization and standard scalar are the common techniques used in preprocessing to rescale the values of features of the fixed range, typically [0,1]. It benefits ML and DL models sensitive to the scale of input features, including RNN-based models. The normalization method ensures that all the features contribute equally to the model, preventing features with large ranges from dominating those with smaller ranges. This process can significantly improve the IDS model performance.

1 $$\:\:\:\:\:\:\:\:\:\:\:X^{\prime\:}=(x-\text{m}\text{i}\text{n}(x\left)\right)/(\text{m}\text{a}\text{x}(x)-\text{m}\text{i}\text{n}(x\left)\:\right)$$

The mathematical formula of the min-max scaler is expressed in Eq. (1). The variable $\:x\:$indicates the input feature values.

2 $$\:\sigma\:=\sqrt{\frac{1}{N}{\sum\:}_{i=1}^{N}{\left({x}_{i}-\mu\:\right)}^{2}}$$

The mathematical formula for the standard deviation ($\:\sigma\:)\:$Scaler is in Eq. (2). The variable $\:{x}_{i}$ ith feature value from the input instances and the notation $\:\mu\:$ indicate the feature’s mean value. The min-max and standard scalar normalized datasets are input to the PSO-optimised GAN and IDS model to strengthen the model’s performance.

## PSO-GRUGAN-IDS proposed model for attack detection in SDN

A strong framework for predicting attacks and identifying anomalies in traffic is developed by the PSO-GRUGAN-IDS model, which combines several technologically advanced approaches. The intrusion detection system (IDS) examines network data to find abnormalities that can point to an attack using the temporal pattern recognition skills of GRU and the optimal settings from PSO. The synthetic data produced by the GAN contributes to improving the training procedure, strengthening the IDS’s resistance to different kinds of attacks.

The entire functionalities of each stage of the PSO-GRUGAN-IDS model are discussed in the following sub-sections. The PSO-GRUGAN-IDS model offers a potent and effective method for predicting attacks and identifying irregularities in network traffic by including these advanced techniques.

### Deep generative adversarial network

Using the GAN model to handle the imbalance data error in the dataset. Using the discriminator, the GAN model can augment the synthetic samples to distinguish the attack samples effectively. The GAN uses a generator (GR) and discriminator (DR) model to efficiently handle the imbalance error. The GR is used to generate synthetic attack data, and the DR model distinguishes the real attack samples by determining the synthetic samples. Refine the GAN model and fine-tune its parameters (including weight updating) to improve the quality of synthetic samples and the effectiveness of attack detection. Integrate the GAN-based attack detection system with the traditional intrusion detection system (IDS) to provide comprehensive network security for the SDN network. By leveraging the power of deep GAN, we can develop robust and adaptive instruction detection or attack forecasting systems capable of predicting sophisticated attacks and anomalies in SDN network traffic, thereby enhancing the overall security posture of the network. So, this research uses Deep GAN to identify the anomalies or attacks in SDN using the InSDN dataset.

### Training the generator (GR) and discriminator (DR) of the GAN model

The twin or parallel network contain generative and discriminative networks. The GR network creates the synthetic samples. It is generated from the real attack samples by taking random samples. The DR network functionality differentiates the synthetic samples generated by the generator. The DR assigns a maximum possibility to real data and a minimum possibility to the GR-generated synthetic data. The GAN model simultaneously discards synthetic data using the gradient information given by the DR.

The attack data is taken from the real attack data distribution, $\:{q}_{data}$, and$\:{\:q}_{g}$ is the GR’s distribution over attack data, and synthetic data vector z comes from a priority distribution $\:{q}_{z}$. The GR takes hidden vector z as input and output to a sample GR (z) to bring GR (z) as close as possible to GR (s). DR is simply a classifier in which $\:DR\:\left(s\right)=1$ if $\:s\sim{q}_{data}$ and $\:DR\:\left(s\right)=0$ if $\:s\sim{q}_{g}$.

3 $$\:\underset{{\theta\:}_{{GR}}}{\text{min}}\underset{{\theta\:}_{{DR}}}{\text{max}}V\left(GR,DR\right)=\:\underset{{GR}}{\text{min}}\underset{{DR}}{\text{max}}{E}_{s\sim{q}_{data}}\left[\text{log}DR\left(s\right)\right]+{E}_{z\sim{q}_{z}}\left[\text{log}\left(1-DR\left(GR\left(z\right)\right)\right)\right]$$

A min-max binary cross entropy objective function is used to train GR and DR models jointly, as in Eq. (3). The *V*(*GR*, *DR*) indicates the binary cross entropy function. GR and DR models use the loss function to back-propagate the model while training it through their respective model update parameters.

$$\:\{\theta\:\_DR^(t+1),\:\theta\:\_GR^(t+1\left)\:\right\}\leftarrow\:\left\{\right(update\:if\:DR\left(x\right)\:\:\:\:\:\:forecasting\:is\:wrong\:$$

$$\:update\:if\:DR\left(GR\left(z\right)\right)\:\:\:\:forecasting\:is\:wrong$$

4 $$\:update\:if\:DR\left(GR\right(z\left)\right)\:\:\:\:\:\:forecasting\:is\:correct$$

The update rule in Eq. (3) is changed as in Eq. (4). The Eq. (4) uses the update parameters of GR ($\:\theta\:\_GR^(t+1))$ and DR ($\:\theta\:\_DR^(t+1))$ at $\:{t}^{th}$Iteration. GR and DR models use the loss function to back-propagate the model while training it through their respective model update parameters.

### PSO for GANs weight initiation

As an alternative to its remarkable success in creating real attack data and training, the performance of GAN remains challenging for various factors: convergence trouble, vanishing gradients, and hyper-parameters optimization. Weight optimization is one of the crucial parts of the DL model. The un-matching weight parameter values create impacts on the models’ overall performance (training time, increased prediction loss rate. So, this study adopts the PSO algorithm to initialize the GAN’s weight initialization. It is crucial to assign suitable weight to train the model to reduce the time and impact, improving accuracy. The PSO identifies the global best (global optima) and assigns it as a weight parameter initial value.

**Fig. 3:**
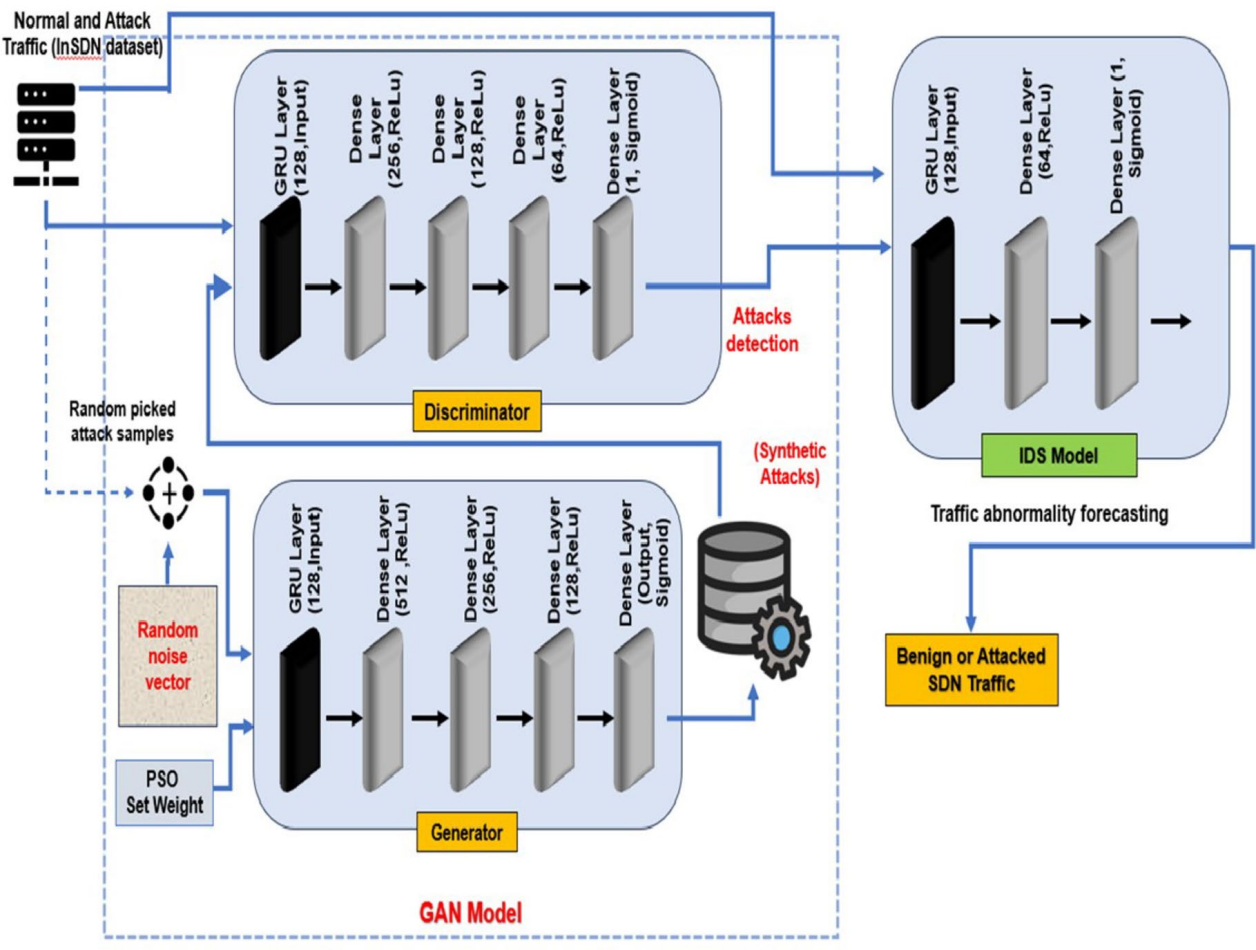
Architecture of proposed PSO-GRUGAN-IDS detection model for traffic abnormality.

PSO is a population-based stochastic optimization technique; it mimics the social behavior of a bird or fish swarm. PSO is characterized by its simplicity, ease of implementation, and ability to handle non-linear and multimodal optimization problems. The PSO is successfully applied to various optimization problems, including feature selection, scheduling, control problems (in engineering and operation research), and parameter turning (including weight, Optimization) in ML and DL. Figure 3 illustrates the architecture of the PSO-optimized GRU-integrated GAN-IDS model for attack detection.

So, this research uses the PSO to optimize the weight parameter of GAN. The effectiveness of PSO lies in its ability to balance exploration (searching the solution space broadly) and exploration (focusing on promising regions) to efficiently find near-optimal solutions in complex optimization landscapes. The aim of the optimization is minimizing or maximizing input variable (X) depending on fitness function f(X). The fitness function evaluates the position vector and determines how good or bad the X = [x_1_, x_2_, x_3_, . x_n_ ] variable. The PSO represents position vector as X_i_^t^=(x_i1_), (x_i2_), (x_i3_) …, (x_in_^T^). The swarm particle (P) is used to determine the number of input samples for the variable (i = 1,2,3, …, P) of the position vector and velocity vector V_i_^t^ = (v_i1_), (v_i2_),(v_i3_),…, (v_in_^T^). The variable t indicates the iteration of each particle, and the variable n indicates the dimensions of samples j = 1,2,3,…,n.

Each particle enticed and randomly moved towards current ($\:{p}^{{best}_{ij}}$) and then personal ($\:{g}^{{best}_{j}}$) global best positions. Each particle updates the current position whenever it finds a better position than the previous one and considers the updated position as the current best position. The objective of the search is to identify the global best among all the current best solutions. The search process continues till no more improvement after a specific iteration.

5 $$\:{V}_{ij}^{t+1}={\omega\:V}_{ij}^{t}+{c}_{1}{r}_{1}^{t}\left[{p}^{{best}_{ij}}-{X}_{ij}^{t}\right]+{c}_{2}{r}_{2}^{t}\left[{g}^{{best}_{j}}-{X}_{ij}^{t}\right]$$

The Eq. (5) forms a new velocity vector $\:{\:\:V}_{ij}^{t+1}$. The two random vectors’ (r_1_ and r_2_) values can be assigned between the range [0,1]. The learning parameters (c_1_ and c_2_) are initialized as c_1_ ≈ c_2_ ≈ 2. The starting stage particle positions are uniformly distributed, and the starting stage of velocity is assigned as (v_i_^(t=0)^ =0). The exploration parameter (inertia weight constant) ω is used to balance the global search, and the ω value must always be higher for the global search. The local search(exploitation) value must be set as low always. This random parameter plays a significant role, as it avoids premature convergences, increasing the most likely global best(optima).

6 $$\:{X}_{ij}^{t+1}={X}_{ij}^{t}+{V}_{ij}^{t+1}$$

In Eq. (6), each particle’s new positions are updated for every iteration. Although $\:{V}_{ij}$ can be any value, it is usually bounded in some range$\:\left[0,{\:V}_{max}\right]$he optimization logic searches for minimums and assesses all position vectors by the fitness function (). This research uses global optima obtained using the PSO algorithm as input to the weight updating parameter value for the GAN classifier to improve and optimize the performance and functions of GAN. By using PSO for weight optimization in deep learning models, the weight space can be effectively searched to find optimal or near-optimal solutions that yield improved performance for the GAN on the intrusion prediction task. This approach is practical when random search methods are impractical due to the high dimensionality of the weight space in the GAN model.

**Algorithm 1:**
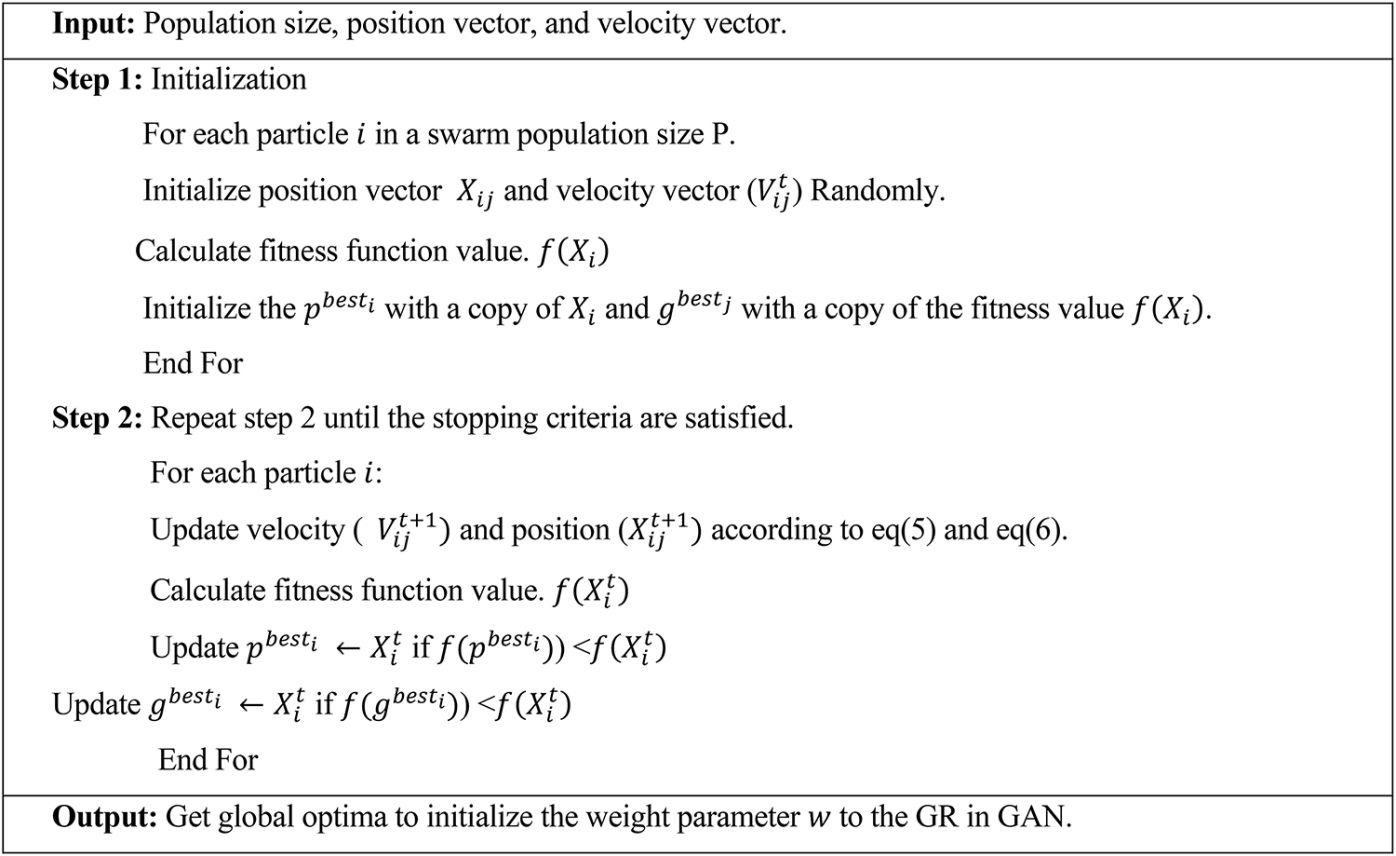
PSO for weight optimization in GAN model.

### GRU layer in generator and discriminator model of GAN

The GRU is one of the popular RNN architectures designed to overcome restrictions in other RNNs. It is specially designed to effectively distinguish temporal dependencies between data (patterns). GRUs are computationally more efficient and can be faster in the training and inference phases. Despite being more straightforward, GRUs are often as effective as LSTM for many sequential data tasks. GRUs are widely used for identifying unusual patterns in time series data, such as network ID. The ID involves applying this type of RNN to analyze and model sequential data patterns typically found in network traffic. So, this study incorporates the GRUs layer in GAN models for network traffic data, making them suitable for detecting time-dependent traffic anomalies.

**Fig. 4:**
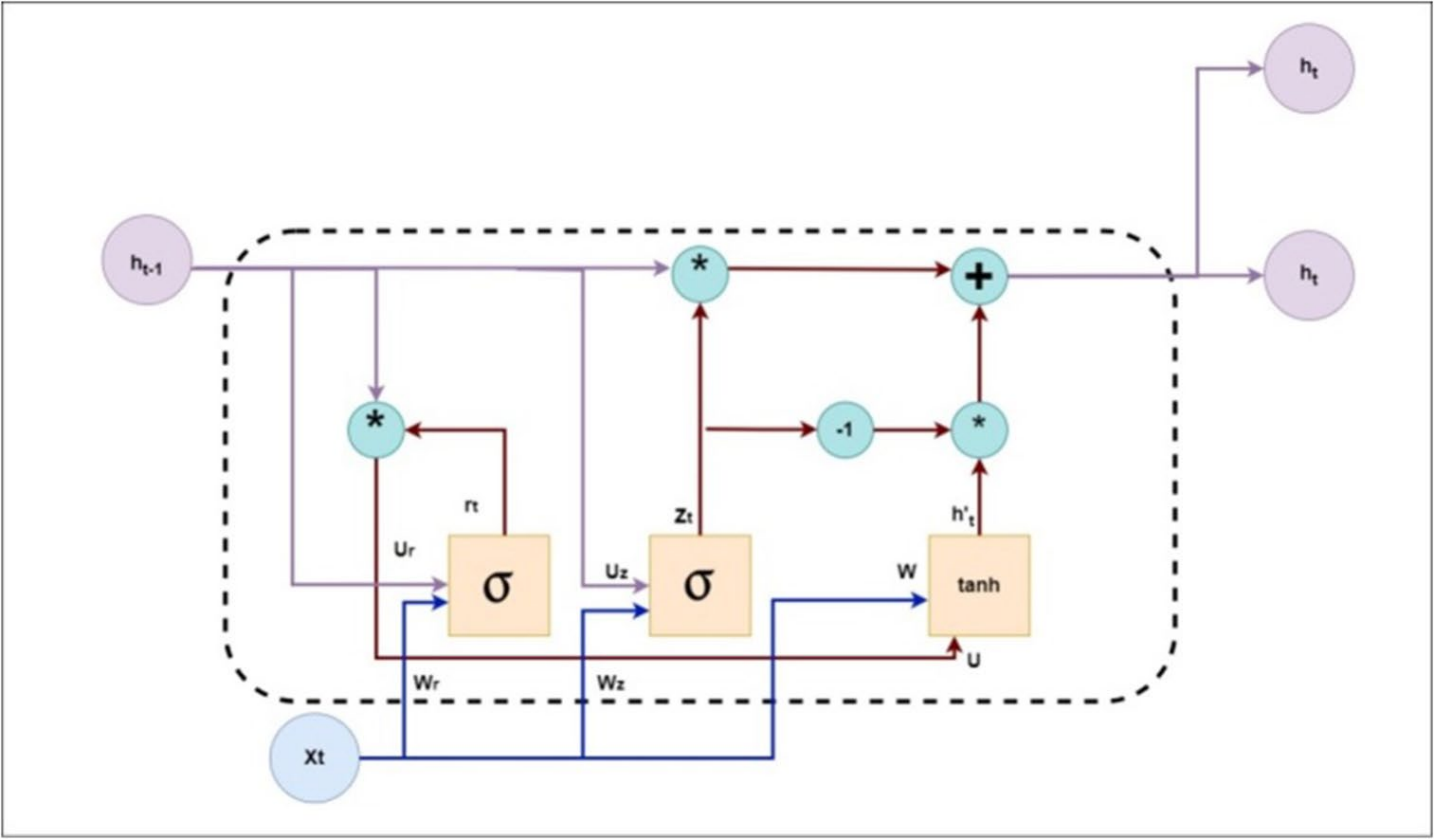
Architecture of the GRU Model^56^.

Figure 4 illustrates the four components and functions of the GRU layer. This layer performs four functions to remember the current input and previously predicted output data: updating the gate, resetting the gate, determining the candidate hidden state (HS), and determining the final HS.

7 $$z_{t} = \sigma \left( {W_{z} ~.~\left[\kern-0.15em\left[ {h_{{t - 1}} ,x_{t} } \right]\kern-0.15em\right]} \right)$$

The mathematical form of the update gate function is represented in Eq. (7). The notation $\:\sigma\:$ indicates the sigmoidal activation function (AF), $\:{W}_{z}$ is the weight matrix for the update gate, $\:{h}_{t-1}$ is the previous HS, and $\:{x}_{t}$ is the current input. The update gate determines the extent to which the HS of the prior time step ($\:{h}_{t-1}$) should be carried forward to the current time step. It supports the GRU in deciding the number of past information required to be remembered.

8 $$r_{t} = \sigma \:\left( {W_{r} \:.\:\:\left[\kern-0.15em\left[ {h_{{t - 1}} ,\:x_{t} } \right]\kern-0.15em\right]} \right)$$

The mathematical form of the reset gate is given in Eq. (8). Its functionalities are similar to those of the update gate. is the weight matrix of the reset gate. It controls how much of the previous HS should be avoided or reset. It allows the model to forget the irrelevant parts of the past state.

9 $$\:\:\:\:\:\:\:\:\:\:\:\:\:\:\:\:\:\:{\stackrel{\sim}{h}}_{t}=tanh\left({W}_{h}\:.\:\:\left[{r}_{t}{\odot}\:{h}_{t-1},\:{x}_{t}\right]\right)$$

The mathematical representation of the candidate HS is expressed in Eq. (9). The notation $\:⨀$indicates the element-wise multiplication, $\:tanh$ is the hyperbolic tangent activation function, and $\:{W}_{h}$ is the weight matrix of the candidate HS. It is a potential new state that could be integrated into the final new HS for the current time step. The reset gate influences it.

10 $$\:{h}_{t}=\left(1-{z}_{t}\right) {\odot} {h}_{t-1}+\:{z}_{t}\: {\odot} {\stackrel{\sim}{h}}_{t}$$

The mathematical derivation of the final HS is expressed in Eq. (10). The final HS for the current time step is computed by combining the previous and candidate HSs, weighted by the update gate.

**Algorithm 2:**
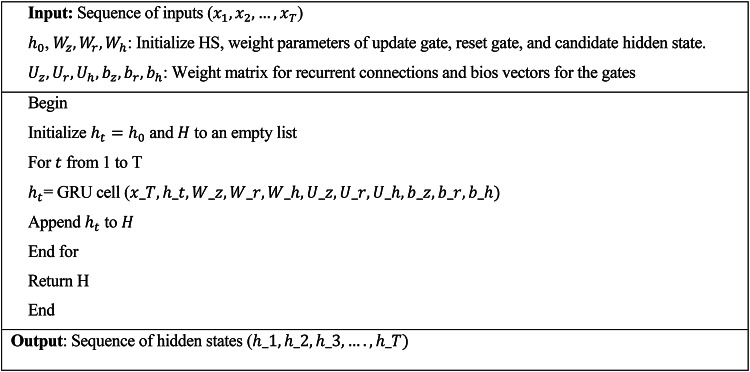
GRU cell layer.

GRUs are particularly well suited for this ID because they can capture temporal dependencies and attack patterns over time. So, this research integrates the GRU layers with GANs GR and DR models to improve the GR’s performance while generating synthetic data and the discriminator’s classification performance while classifying the synthetic data effectively. This research integrates the GRU layer in the GR and DR of the GAN model to improve the model’s detection accuracy.

### Activation functions

The Rectified Linear Unit (ReLU) AF is suitable for performing the GAN model’s state activation decisions.

11 $$\:\:\:\:\:\:\:\:\:\:\:\:ReLU\left(\text{max}\left(0,p\right)\right)$$

12 $$\:{ReLU}^{{\prime\:}}\left(p\right)=\left\{\begin{array}{c}1\:\:\:\:\:\:p>0\\\:0\:\:\:\:\:p<0\end{array}{\forall\:}_{p}=-1,\dots\:,1\right.$$

13 $$\:Sigmoid\:\left(x\right)=1/\left(\right(1+e^(-x)\:\left)\:\right)$$

The representation of the ReLU AF is given in Eq. (11), and the state activation condition of ReLU is shown in Eq. (12). The variable denotes the forecasted value. Whenever the computed hidden node values and weights achieve 1, the ReLU activates the gate to train the sample. The ReLU considers − 1 the minimum loss rate to allow backpropagation and update node weights. This gate helps forecast correct attack data. The sigmoidal AF is given in Eq. (13) for the final attack classification.

**Algorithm 3:**
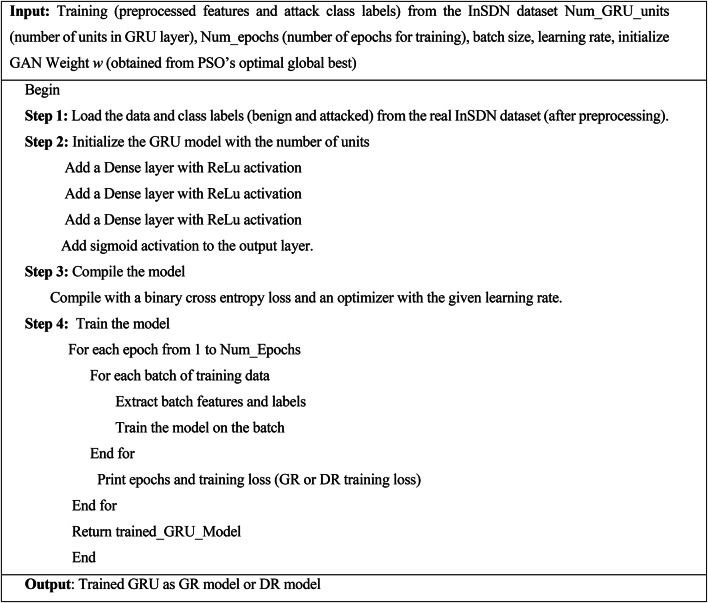
GRU Layer integrated in GAN model.

The PSO-optimized GRU-based GAN model generated attack data, and the real InSDN attack data are utilized to evaluate the GRU-based IDS model. The methodology and functions of the IDS model are given in the subsequent section.

### GRU-driven IDS model for anomaly classification

GRUs are computationally efficient compared to LSTMs, making them suitable for real-time IDS. GRUs can handle sequences of varying lengths and are robust against the vanishing gradient problem. IDS aims to identify unauthorized access or anomalies that may indicate malicious activities.

GRU architecture contains four layers, including the input layer, the GRU layer, the Dense layer, and the output layer. Define the input shape based on the number of features and the length of the sequences. Stack one or more GRU layers. More layers can help capture complex patterns but may require more computational resources. A dense layer (fully connected) to map the GRU outputs to the desired number of output classes (normal or attack). Use a sigmoidal AF for binary classification. The IDS model also uses the GRU layer to forecast the network abnormality by remembering the previous output and current input.

**Algorithm 4:**
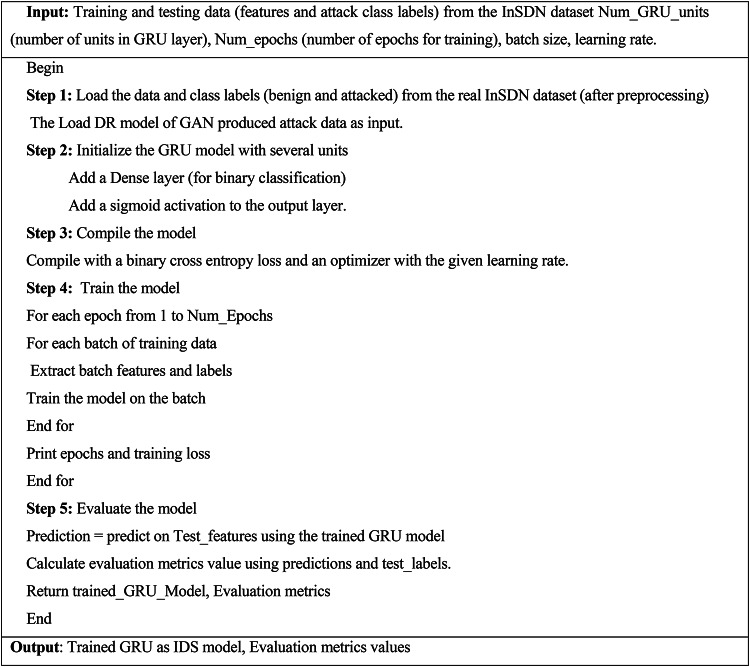
GRU-IDS model for SDN traffic classification.

**Algorithm 5:**
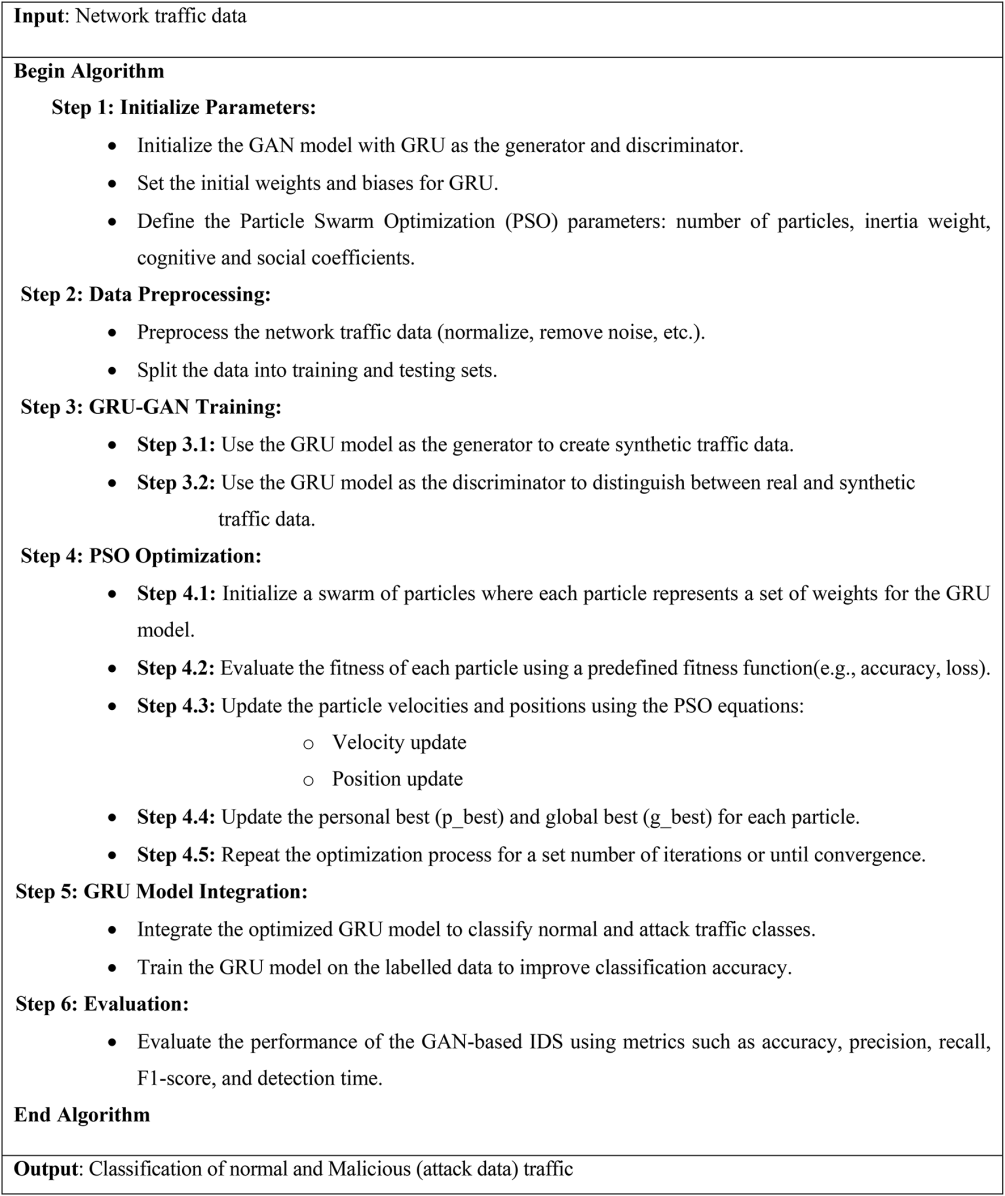
Overall proposed PSO-GRUGAN-IDS model (GAN-based IDS with PSO optimization) for identifying anomalies in SDN traffic.

The algorithm begins by initializing the parameters necessary for setting up the GAN model, where a GRU model functions both as the generator and discriminator. The initial weights and biases for the GRU are configured, and the Particle Swarm Optimization (PSO) parameters, including the number of particles, inertia weight, and cognitive and social coefficients, are defined to optimize the model’s performance.

Next, the network traffic data undergoes preprocessing, which involves normalizing the data and removing any noise to ensure clean input for model training. The data is then split into training and testing sets to facilitate both the learning and evaluation phases of the model.

During the GRU-GAN training phase, the GRU model first acts as a generator to produce synthetic attack traffic data that mirrors the traffic patterns. Simultaneously, the GRU model serves as a discriminator to differentiate between real and synthetic attack data, which helps in refining the model’s ability to detect anomalies. PSO optimization follows, where a swarm of particles is initialized, each representing a potential set of weights for the GRU model. The fitness of each particle is assessed using metrics such as accuracy and loss, guiding the optimization process. The particles update their velocities and positions based on the PSO equations, which factor in both personal and global best positions. This iterative process continues until a set number of iterations is completed or convergence is achieved, leading to an optimized GRU model.

Once optimized, the GRU model is integrated into the IDS framework to classify network traffic as either normal or malicious (attack data). This model is further trained using labeled data to enhance its classification accuracy. Finally, the performance of the GAN-based IDS is evaluated using metrics such as accuracy, precision, recall, F1-score, and detection time, ensuring the system’s effectiveness in detecting network threats and anomalies.

The trained PSO-GRUGAN-IDS model is designed to identify the different attacks in the 5G SDN layers. This model can be deployed in the control plane of an SDN network to manage network activities and control traffic. The PSO-GRUGAN-IDS detects traffic abnormalities by continuously monitoring the SDN network traffic activity data. The performance of the PSO-GRUGAN-IDS is discussed in a subsequent section.

## Result and discussions

This section discusses the performance analysis of the PSO-GRUGAN-IDS model-based traffic abnormality detection. The model is implemented and evaluated using Python Tensorflow libraries with many prebuilt ML and DL-based model functions. The efficiency of the model is estimated by comparing the performance of the PSO-GRUGAN-IDS model with the existing DL-based intrusion detection approaches on the InSDN dataset, including GRU-GAN^31^, DNN^32^, PSO-DNN^34^, GAN-LSTM^35^, PSOGRU, and PSO-GAN-LSTM. The comparison models are chosen based on their performance in recent years. This study uses the InSDN dataset to train the PSO-GRUGAN-IDS model and evaluate the model performance using different accuracy metrics such as accuracy, precision, recall, f1-score, specificity, Root mean square error (RMSE), Mean Absolute error (MAE), Log loss, throughput, attack detection time, CPU utilization, ROC cure area, and Evasion increase rate (EIR). In addition, two other datasets EDGE_IIoT and BoT-IoT are used to evaluate the proposed model, demonstrating its high accuracy in the context of 5G SDN networks. These datasets further validate the model’s performance.

The dataset is designed and labeled for training and testing the SDN environment’s intrusion detection system (IDS). The generator (GR) model takes 10,000 attack data as input to generate a synthetic attack, and the GR model generates 10,000 synthetic attack data and 10,000 real attack data. Finally, the IDS model has taken 20,000 attack data (Real attack and synthetic) from the PSO-GAN Model and 10,000 attack data from the InSDN dataset and uses 30,000 Benign data from the InSDN dataset for the training and testing phase (a total of 60,000 data instances) to determine the SDN data traffic as attack traffic or benign traffic in 5G SDN networks. Table 2 illustrates the Hyperparameter values for PSO optimization, GAN model and IDS used by the PSO-GRUGAN-IDS model for traffic abnormality detection.

**Table 2:** Hyperparameter values used by the PSO-GRUGAN-IDS model for traffic abnormality detection.

Parameters	Value
PSO optimizer	
Number of particles	10
Convergence threshold	0.5
Inertia weight	0.5
Cognitive weight	0.8
Social weight	0.8
Global best fitness value	Dynamic (converges during optimization)
Iteration count	Dynamic (depends on convergence)
GAN model	
GR layers	GRU layer (128 units)
	Dense (512 units, relu)
	Dense (256 units, relu)
	Dense (128 units, relu)
	Dense(output_dim, sigmoid)
GR optimizer	Adam
learning_rate	0.0005
beta_1	0.5
DR layers	GRU (128 units)
	Dense (256 units, relu)
	Dense (128 units, relu)
	Dense (64 units, relu)
	Dense (1 unit, sigmoid)
DR optimizer	Adam
learning_rate	0.0002
beta_1	0.5
Discriminator loss function	Binary cross-entropy
Combined model loss function	Binary cross-entropy
GAN training epochs	25
GAN batch size	32
IDS model	
Model layers	GRU(128 units)
	Dense(64 units, relu)
	Dense(1 unit, sigmoid)
Optimizer	Adam
learning_rate	0.0005
beta_1	0.5
The loss function	Binary cross-entropy
Epochs	25
Batch size	32

**Fig. 5:**
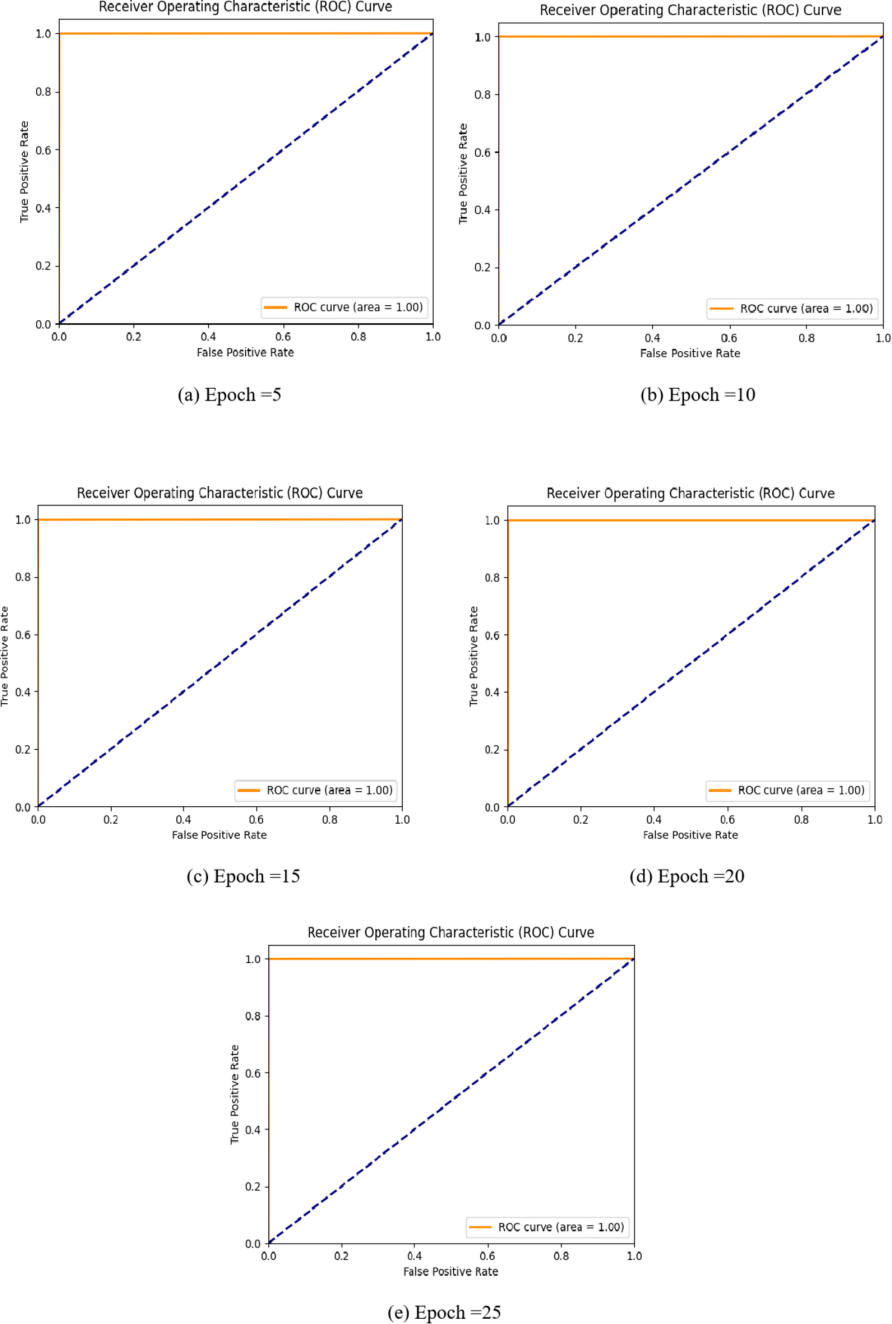
ROC curve of PSO-GRUGAN-IDS model for threat identification.

The Fig. 5. demonstrates the ROC curve obtained for five epochs. The best ROC curve for traffic attack detection should exhibit a high True positive rate (TPR) and a lower false positive rate (FPR). The area under the ROC curve represents the performance. The higher the ROC value, the closer to 1. The results show that the PSO-GRUGAN-IDS achieves ROC values closer to 1 for each epoch.

The PSO-GRUGAN-IDS model’s functionality is assessed by examining its results against the outcomes of other deep learning-based intrusion detection techniques like PSO-GRU, GRU-GAN^31^, DNN^32^, PSO-DNN^34^, GAN-LSTM^35^, and PSO-GAN-LSTM using the InSDN dataset for different performance metrics. Table 3 illustrates the comparison between deep learning-based intrusion detection techniques against the proposed work of the PSO-GRUGAN-IDS Model for different numbers of Epochs for different classification metrics like Accuracy, Precision, Recall, F1-Score, and Specificity with a specific higher rate value of vulnerability and malicious traffic detection in 5G Software Defined Networks. Training time and Detection time were achieved at a lesser rate for rapid detection of attacks. Table 4 highlights a comparison of the projected PSO-GRUGAN-IDS Model work for varying numbers of epochs for different Evaluation metrics with deep learning-based intrusion detection approaches. The Evaluation metrics are Root mean square error, Mean Absolute error, Log loss rate, Throughput, CPU utilization in which the error function attains the minimum value. A low root mean square error (RMSE) provides more accurate predictions. Where CPU Utilization also decreases, indicating a decrease in the amount of CPU processing power needed for 5G SDN Network threat identification. The throughput of this overall work hooks the greater value where more samples can be generated per second.

**Fig. 6:**
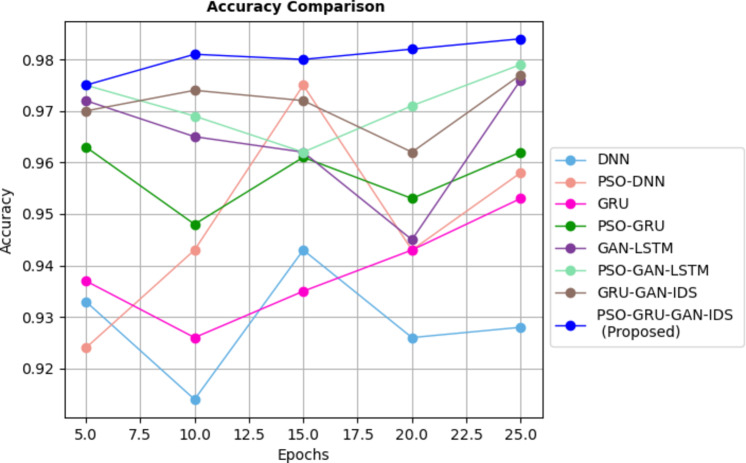
Accuracy rate comparison for different DL Models with InSDN dataset.

The remarkable performance is shown in Fig. 6. of various attack detection models for the InSDN dataset. Notably, the PSO-GRUGAN-IDS model stands out with a maximum accuracy rate of 0.984, surpassing other models such as DNN, PSO-DNN, GRU, PSO-GRU, GAN-LSTM, PSO-GAN-LSTM, and GRU-GAN-IDS this exceptional performance of the model’s potential as a superior IDS framework for 5G SDN network.

**Table 3:** Comparison of deep learning models for different classification metrics using InSDN dataset.

Classification metrics	No. of epochs	DL Models for different classification metrics using InSDN dataset							
		DNN	PSO- DNN	GRU	PSO-GRU	GAN-LSTM	PSO-GAN-LSTM	GRU-GAN-IDS	PSO-GRUGAN-IDS (proposed work)
Accuracy	5	0.933	0.924	0.937	0.963	0.972	0.975	0.970	0.975
	10	0.914	0.943	0.926	0.948	0.965	0.969	0.974	0.981
	15	0.943	0.975	0.935	0.961	0.962	0.962	0.972	0.980
	20	0.926	0.943	0.943	0.953	0.945	0.971	0.962	0.982
	25	0.928	0.958	0.953	0.962	0.976	0.979	0.977	0.984
Precision	5	0.91	0.929	0.935	0.943	0.961	0.961	0.979	0.98
	10	0.915	0.925	0.935	0.945	0.965	0.964	0.975	0.979
	15	0.924	0.933	0.944	0.953	0.96	0.97	0.978	0.978
	20	0.925	0.935	0.945	0.955	0.965	0.978	0.981	0.979
	25	0.938	0.945	0.952	0.962	0.971	0.981	0.98	0.98
Recall	5	0.912	0.922	0.932	0.94	0.972	0.981	0.975	0.982
	10	0.915	0.925	0.935	0.945	0.974	0.982	0.976	0.981
	15	0.924	0.932	0.942	0.951	0.973	0.982	0.977	0.985
	20	0.925	0.935	0.945	0.955	0.978	0.979	0.978	0.98
	25	0.93	0.942	0.952	0.962	0.98	0.981	0.98	0.984
F1-score	5	0.912	0.924	0.935	0.942	0.966	0.971	0.977	0.981
	10	0.915	0.925	0.935	0.945	0.969	0.973	0.975	0.98
	15	0.926	0.936	0.943	0.951	0.966	0.976	0.977	0.981
	20	0.925	0.935	0.945	0.955	0.971	0.978	0.979	0.979
	25	0.938	0.944	0.957	0.965	0.975	0.981	0.98	0.982
Specificity	5	0.91	0.915	0.923	0.932	0.952	0.958	0.962	0.984
	10	0.915	0.921	0.931	0.944	0.963	0.969	0.973	0.982
	15	0.922	0.932	0.942	0.956	0.968	0.971	0.978	0.98
	20	0.925	0.943	0.951	0.965	0.971	0.975	0.979	0.983
	25	0.931	0.945	0.961	0.977	0.975	0.978	0.979	0.986
Root mean square error (RMSE)	5	7.53	4.63	4.45	4.23	4.14	3.92	1.92	1.78
	10	5.56	3.76	3.53	3.42	3.31	3.01	2.63	2.34
	15	4.12	3.92	3.86	3.74	3.61	3.5	2.51	1.2
	20	6.53	4.65	2.51	2.49	2.43	2.36	2.24	1.82
	25	5.54	3.96	2.92	2.85	2.73	2.54	1.91	1.74
Mean absolute error (MAE)	5	5	4.3	3.7	3.9	2.8	2.2	2.3	2.1
	10	5.2	4.7	4.8	3.8	2.5	2.1	2	1.9
	15	4.5	3.4	5.4	5.1	2.8	2	1.9	1.6
	20	6.6	5.8	4.3	5.2	2.4	2	2.2	1.8
	25	5.5	5.7	4.2	4.5	2.4	1.9	2.3	1.7

**Table 4:** Comparison of deep learning models for different evaluation metrics using InSDN dataset.

Evaluation metrics	No. of epochs	Deep learning models for different evaluation metrics using InSDN dataset							
		DNN	PSO DNN	GRU	PSO-GRU	GAN-LSTM	PSO-GAN-LSTM	GRU-GAN-IDS	PSO-GRUGAN-IDS (proposed work)
Training time (sec)	5	200.34	695.38	490.34	485.29	380.24	359.55	278.35	185.1
	10	440.31	525.23	413.21	385.86	480.65	379.86	379.23	279.71
	15	606.77	719.67	590.92	575.38	650.34	579.93	679.67	352.81
	20	823.19	705.88	790.34	785.47	780.46	779.92	779.25	503.82
	25	998.35	995.13	990.29	985.28	980.46	979.63	879.85	564.67
SDN traffic detection time (sec)	5	3.954	3.452	2.351	2.475	2.998	2.872	2.845	2.474
	10	4.035	3.947	3.524	2.769	3.538	3.484	3.357	2.478
	15	4.145	4.174	4.345	3.456	4.335	3.545	3.134	2.464
	20	4.557	4.285	4.679	3.646	4.855	4.193	4.035	3.444
	25	4.679	4.735	4.936	4.346	5.643	4.483	4.457	3.465
Log loss rate	5	5.5	5.4	4.3	4.2	4.1	3.2	2.9	1.75
	10	4.5	5.4	4.8	4.3	4.1	2.1	2.4	1.6
	15	5.9	5.2	4.9	4.7	3.6	2.6	2.4	1.59
	20	4.2	4.15	4.1	3.5	3.1	3.4	1.9	1
	25	4.5	4.2	4.4	3.35	3.3	3.25	2.2	1.2
Throughput (samples/sec)	5	2000	2100	2200	2300	2350	2400	2430	2443.66
	10	1800	1900	2000	2100	2150	2200	2230	2251.33
	15	1850	1900	2000	2100	2150	2200	2250	2327.08
	20	1850	1900	2000	2100	2150	2200	2250	2327.05
	25	1900	2000	2100	2200	2250	2300	2325	2567.89
CPU utilization (%)	5	14.90	14.35	12.45	12.34	8.81	9.24	10.43	8.33
	10	14.43	15.78	13.57	10.46	8.75	9.14	12.57	6.80
	15	14.78	15.89	14.69	13.48	9.88	7.78	8.36	4.45
	20	15.22	16.33	16.77	15.89	9.28	8.55	8.45	5.48
	25	16.22	15.35	15.67	14.38	10.32	7.57	7.36	4.77

**Fig. 7:**
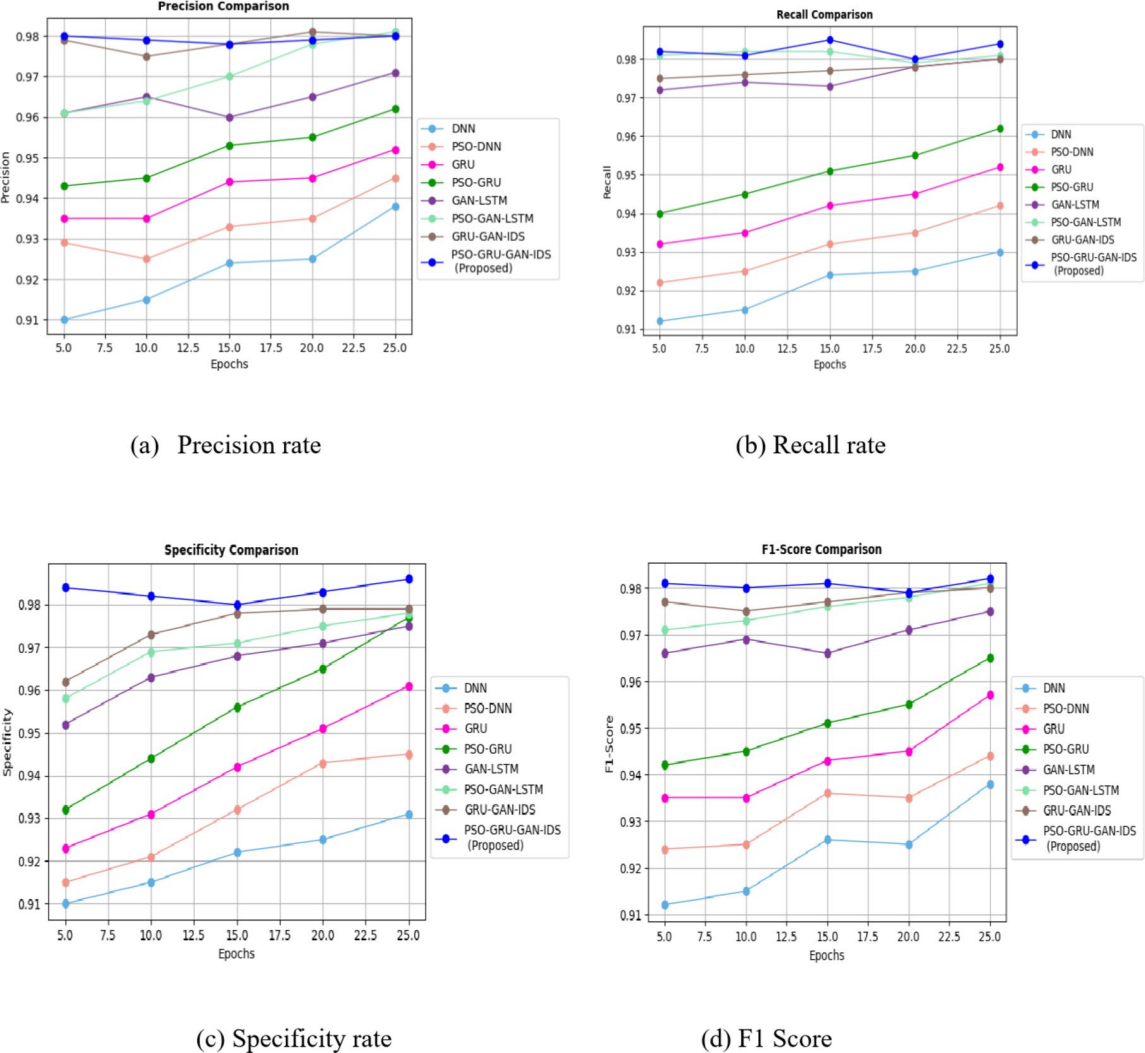
Evaluation of Precision, Recall, Specificity, F1 score for different DL Models with InSDN dataset.

Figure 7 showcases a crucial finding the PSO-GRUGAN-IDS model, with its exceptional precision rate of 0.98, recall rate of 0.985, specificity rate of 0.986, F1 score rate of 0.982 outperforms the other attack detection models such as DNN, PSO-DNN, GRU, PSO-GRU, GAN-LSTM, PSO-GAN-LSTM, and GRU-GAN-IDS. This significant performance difference underscores the importance of our research in network security, particularly in the context of 5G SDN networks.

**Fig. 8:**
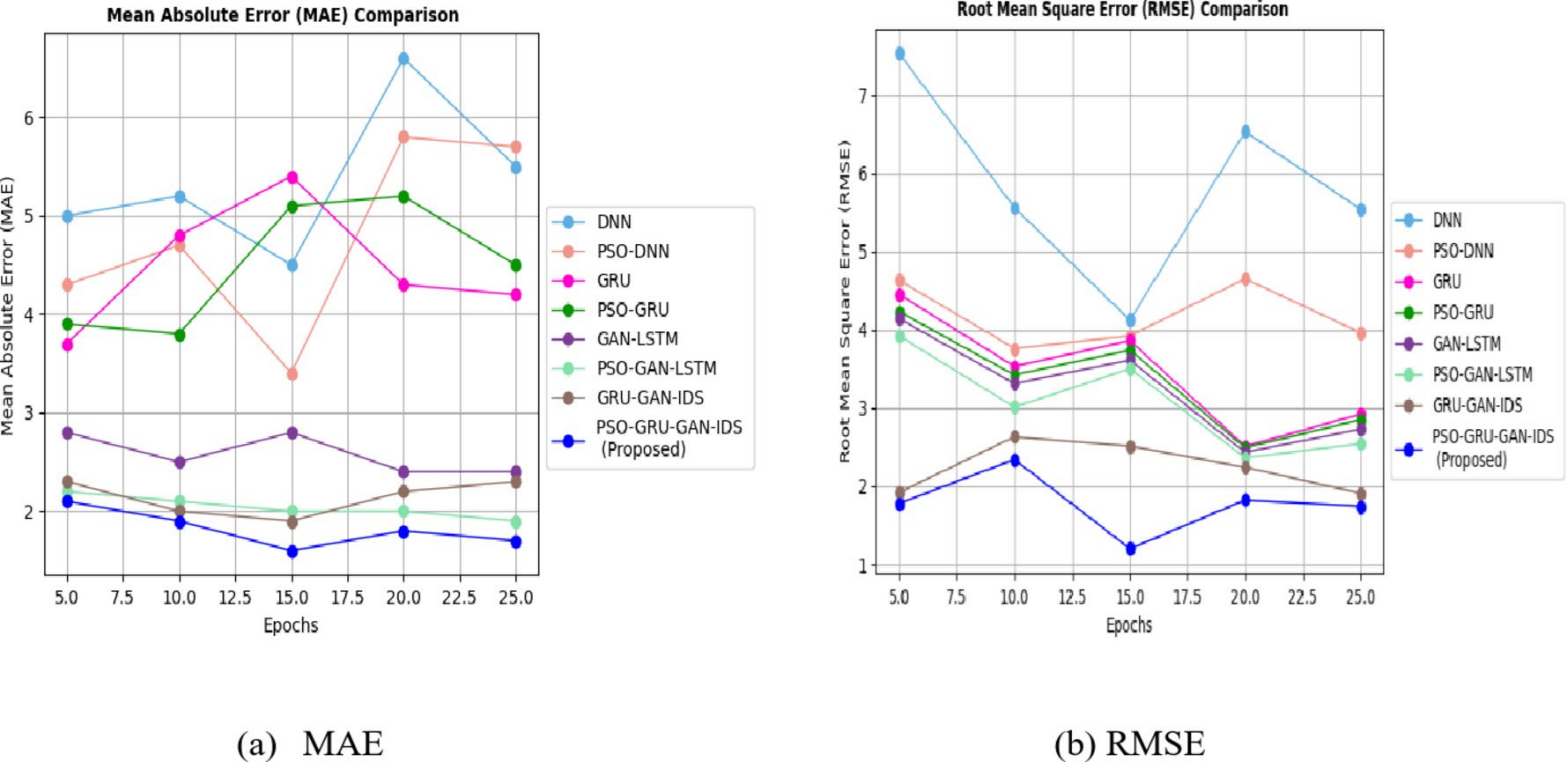
MAE and RMSE comparison for various DL models with InSDN dataset.

Figure 8 demonstrates the MAE and RMSE rate comparison of various attack detection models for the InSDN dataset. The comparison analysis shows that the PSO-GRUGAN-IDS model obtains a minimum MAE rate of 1.6 and RMSE rate of 1.2 which is very low compared to other attack detection models such as DNN, PSO-DNN, GRU, PSO-GRU, GAN-LSTM, PSO-GAN-LSTM, and GRU-GAN-IDS.

**Fig. 9:**
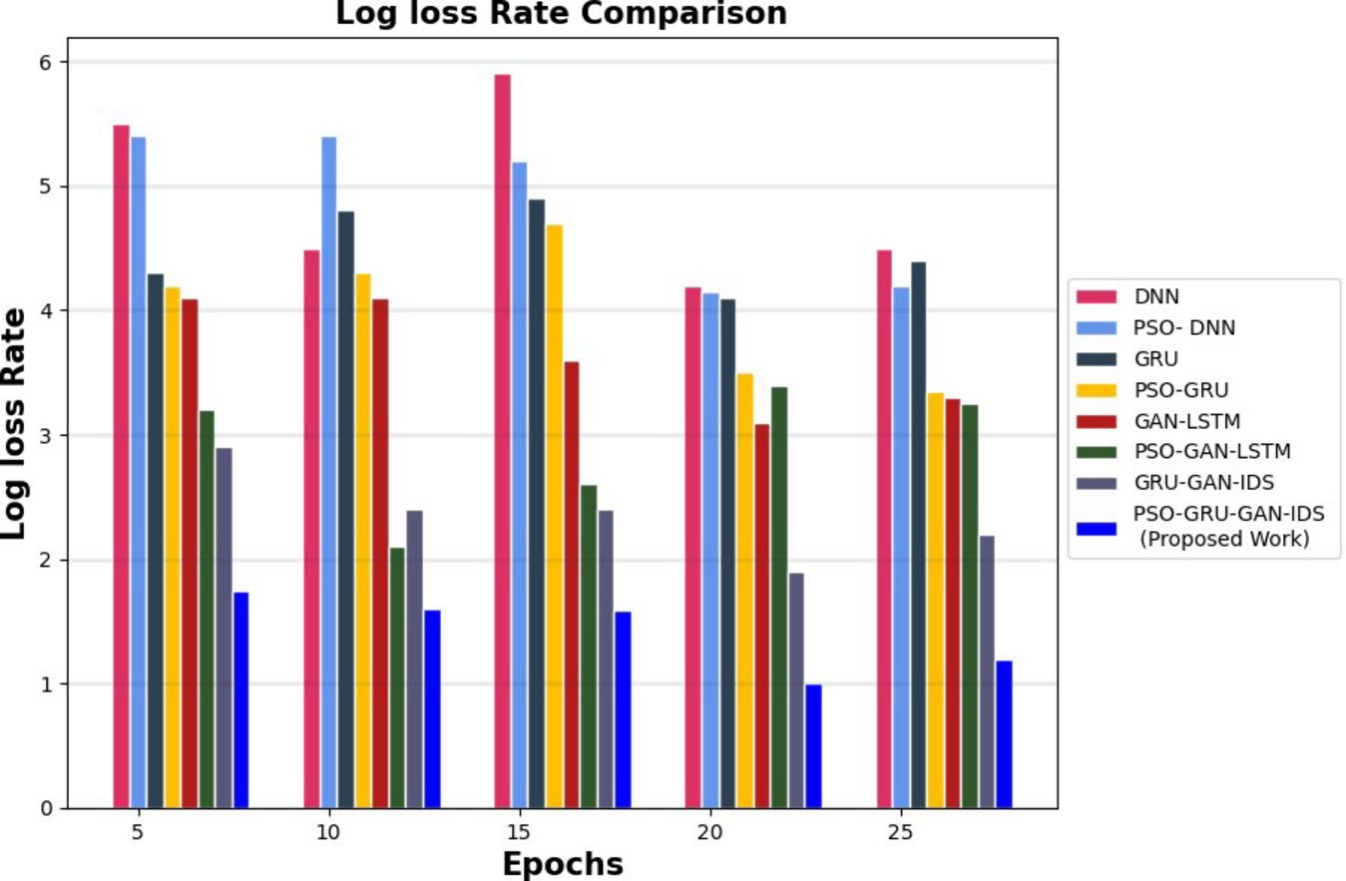
Log Loss rate comparison for InSDN dataset with different DL models.

Figure 9 demonstrates the Log Loss rate comparison of various attack detection models for the InSDN dataset. The comparison analysis shows that the PSO-GRUGAN-IDS model obtains a lesser Log loss rate of 1.0, which is low compared to other attack detection models such as DNN, PSO-DNN, GRU, PSO-GRU, GAN-LSTM, PSO-GAN-LSTM, and GRU-GAN-IDS.

**Fig. 10:**
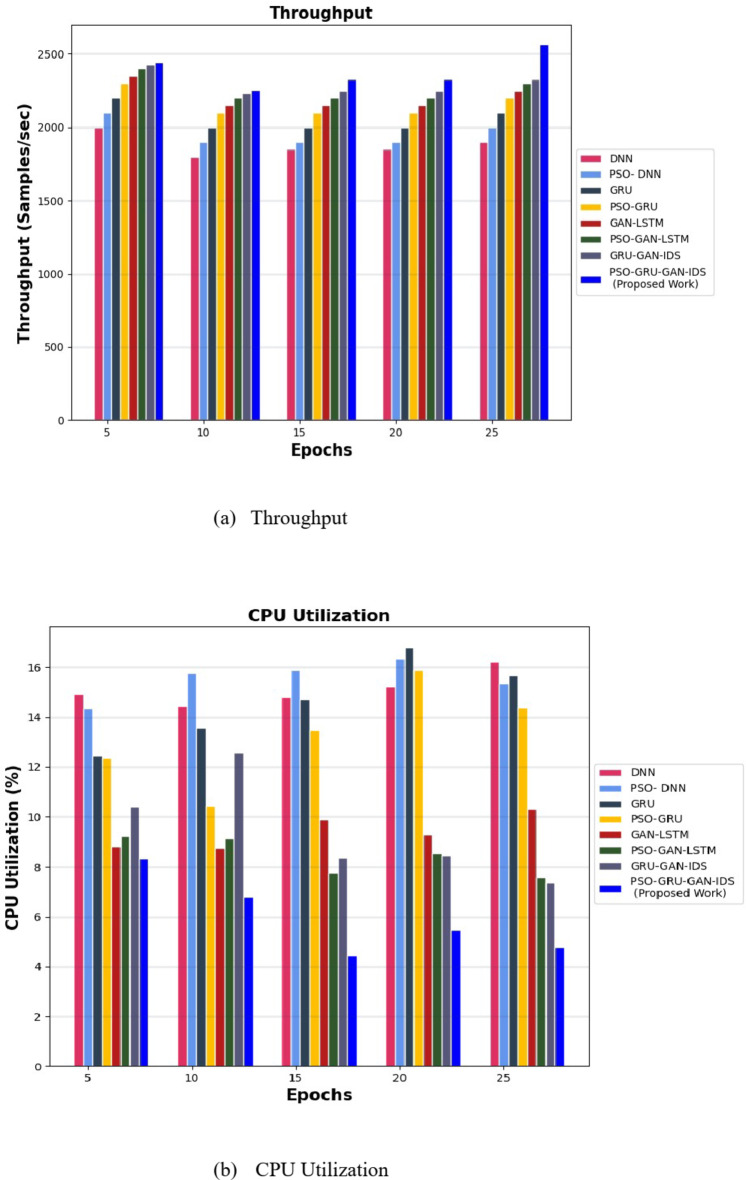
Throughput an CPU Utilization comparison for InSDN dataset with other DL Models.

This demonstrates a Throughput and CPU utilization comparison for various attack detection models for the InSDN dataset in Fig. 10. The comparison analysis shows that the PSO-GRUGAN-IDS model obtains the lowest CPU utilization rate of 4.449% for training the model and attack identification and obtains a higher throughput rate of 2567 samples/seconds for training the model.

**Fig. 11:**
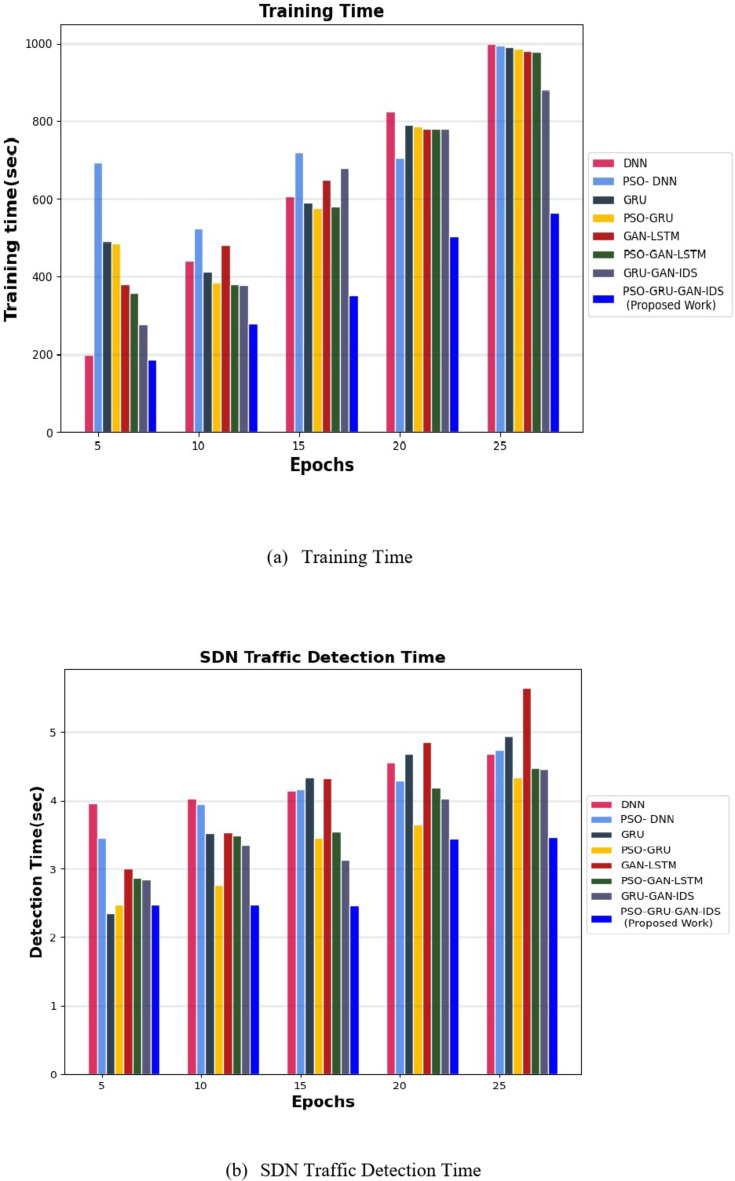
Training time and Detection time comparison for the InSDN dataset with various DL models.

The PSO-GRUGAN-IDS framework integrates PSO with a GAN featuring a GRU layer for IDS in SDN environments. This combination aims to optimize weight initialization, enhance temporal data handling, and improve detection performance in dynamic 5G networks.

### Training time complexity

PSO Component: The PSO algorithm optimizes the initial weights for the GAN. The time complexity of PSO is typically $\:O(P\:\times\:I\times\:D)$, where P is the number of particles, I is the number of iterations, and D is the dimensionality of the search space.

GAN Component: The GAN training involves a generator and a discriminator, each typically with a time complexity of $\:O(T\times\:N\times\:M)$, where T is the number of epochs, N is the number of samples, and M is the number of parameters in the network.

GRU-IDS Component: The GRU layer adds complexity due to its recurrent nature, with each operation within a layer being $\:O(N\times\:{H}^{2})$, where H is the number of hidden units, and N is the input size. Overall, the training time complexity of the PSO-GRUGAN-IDS model can be approximated as

14 $$\:O(P\times\:I\times\:D+T\times\:N\times\:M+N\times\:{H}^{2})$$

### Detection time complexity

Once trained, the detection time primarily involves the forward pass through the GAN’s discriminator and the GRU-IDS layer, with a complexity of $\:O(N\times\:{H}^{2})$, where N is the input size and H is the number of hidden units.

### Scalability analysis

The results in Fig. 11 demonstrate that the PSO-GRUGAN-IDS model achieves the least training time of 185.1 s and a less detection time of 2.464 s when identifying threats in the InSDN dataset. This efficiency highlights the model’s suitability for real-time 5G environments due to:

Fast Training: The optimized weight initialization via PSO reduces convergence time during training, essential for quick deployment in dynamic 5G networks.

Quick Detection: The low detection time ensures that the model can respond to threats promptly, which is critical for maintaining the high throughput and low latency requirements of 5G networks.

Handling Large-scale Data: The use of GANs for data augmentation and GRU for temporal data management allows the model to effectively handle large volumes of data typical in 5G environments, ensuring robustness and reliability in real-time scenarios.

In summary, the PSO-GRUGAN-IDS framework demonstrates favorable time complexity and scalability, making it a viable solution for real-time threat detection in 5G SDN environments, where rapid training and detection are crucial.

**Table 5:** Comparison of detection effectiveness metrics for GRU-GAN and PSO-GRU-GAN model.

Epochs	GRU-GAN model				PSO-GRUGAN (proposed model)			
	GR loss	DR loss	Detection rate	EIR	GR loss	DR loss	Detection rate	EIR
5	2.2	2.68	93.8	-0.1	0.69	0.66	96.9	-0.2
10	3.92	3.64	94.2	-0.05	0.71	0.62	97.9	-0.08
15	3.96	3.96	94.8	-0.7	0.70	0.576	95.87	-0.78
20	2.1	2.954	95.6	-0.05	0.72	0.53	97.43	-0.1
25	3.92	3.51	95.8	-0.1	0.73	0.49	96.22	-0.15

Table 5 contains the attack detection rate, EIR rate, and GR and DR loss of the GRU-GAN and PSO-GRU-GAN models. The GR loss, GR loss, and Detection rate in the table show the stable loss rate in each iteration, resulting in a robust GAN capable of detecting traffic attacks efficiently. The best loss rates for the GR and DR in a GAN model for attack detection are those that reflect a well-balanced training process where both models improve concurrently without dominating one another. The minimum EIR rate for traffic attack detection can enhance the detection capabilities.

**Fig. 12:**
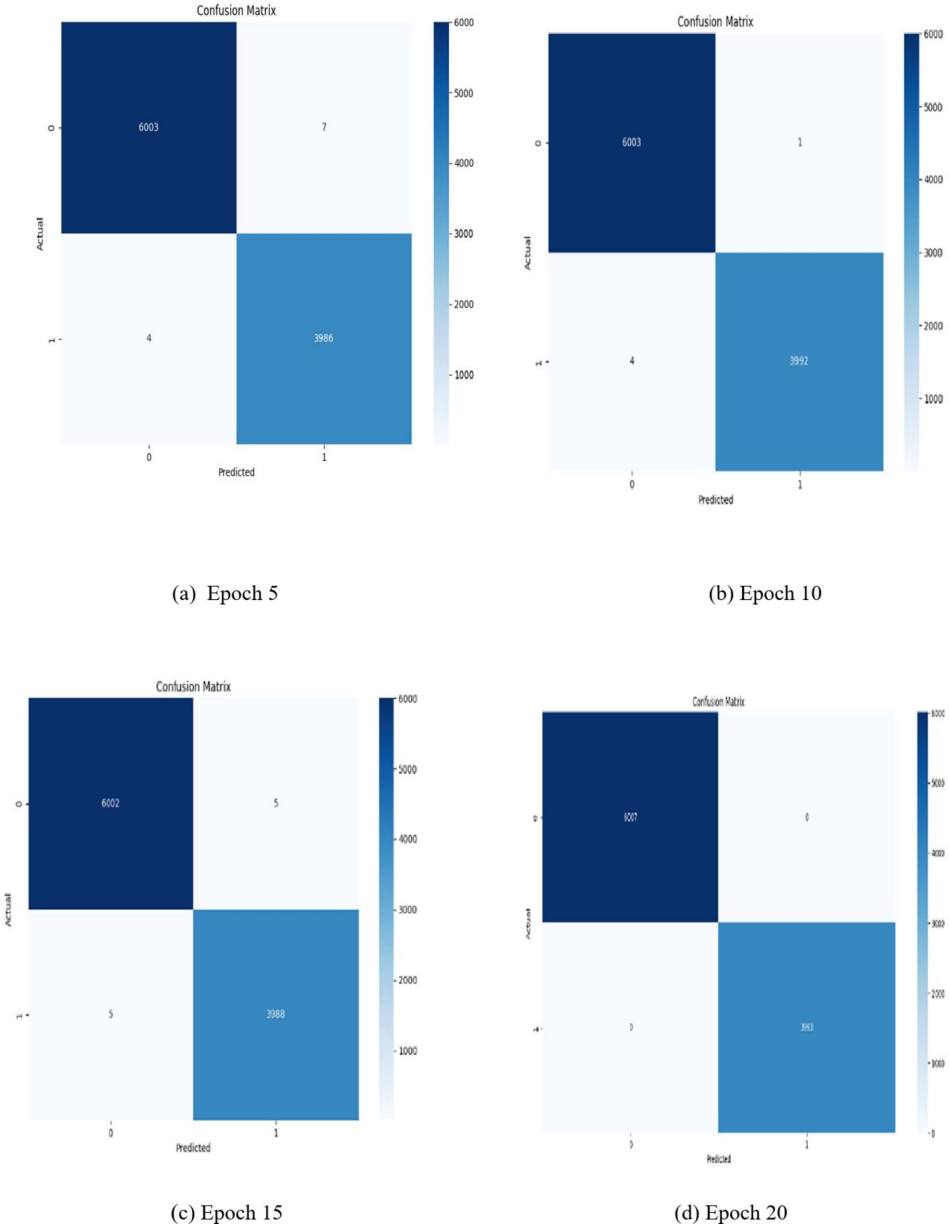
Confusion Matrix generated for first four prosperous epochs.

Figure 12 shows the resultant confusion matrix for the actual and predicted data. It is essential for traffic attack detection as it provides detailed insights into the model’s performance, helps understand errors and guides improvements to enhance security and usability.

**Table 6:** Performance comparison of PSO-GRUGAN-IDS with state-of-the-art methods on traffic attack datasets.

Refs & year	Algorithms	Dataset	Accuracy (%)	Precision (%)	Recall (%)	F1-score (%)
Han et al. (2024)^45^	BPSO-SA- LightGBM	CICDDoS2019	99.96	99.96	99.97	99.95
A. A. E. B. Donkol et al. (2023)^46^	LPPSO- enhanced LSTM	KT-21	96.89	99.93	96.99	98.44
A. A. E. B. Donkol et al. (2023)^46^	LPPSO- enhanced LSTM	KD-P	99.93	99.99	99.96	99.97
Wahab et al. (2024)^47^	BiLSTM + GRU	CICDDoS2019	99. 86	99. 96	99. 903	99.93
Rani et al. (2024)^49^	IChOA-DINet	APA-DDoS Dataset	97	97	NA	NA
Hnamte et al. (2024)^50^	DNN	InSDN	99.98	99.98	99.98	99.98
Hnamte et al. (2024)^50^	DNN	CICIDS2018	100	99.99	99.98	99.97
Hnamte et al. (2024)^50^	DNN	Kaggle DDoS	99.99	99.99	99.97	99.96
Maddu et al. (2024)^51^	ResNet152V2- SMA- DCGAN	Edge IIoT	99.31	99.25	99.21	99.23
Our (proposed work)	PSO-GRUGAN-IDS	InSDN	98.4	98	98.5	98.20
Our (proposed work)	PSO-GRUGAN-IDS	Kaggle EDGE_IIoT	99.98	99.99	99.97	99.98
Our (proposed work)	PSO-GRUGAN-IDS	Kaggle BoT-IoT	99.97	99.98	99.98	99.97

The Table 6 summarizes the performance of various algorithms on different datasets in terms of accuracy, precision, recall, and F1-score. The BPSO-SA-LightGBM algorithm achieves high accuracy of 99.96% on the CICDDoS2019 dataset. The LPPSO-enhanced LSTM shows varied performance on different datasets, with an accuracy of 96.89% on KT-21 and 99.93% on KD-P. BiLSTM + GRU also performs well on CICDDoS2019 with an accuracy of 99.86%. IChOA-DINet and DNN models show high accuracy on APA-DDoS, InSDN, CICIDS2018, and Kaggle DDoS datasets, with DNN reaching 100% on CICIDS2018. The GRU-Attention and ResNet152V2-SMA-DCGAN models are listed without specific performance metrics.

Many of the datasets used in previous work were outdated and unable to handle 5G SDN network traffic in real-time scenarios particularly when it came to adapting to rapid changes in traffic patterns within the 5G SDN network. They struggled to detect unusual packet sizes or a sudden surge in connections from a particular IP address. In contrast, the proposed PSO-GRUGAN-IDS model for 5G networks integrates a GRU, which improves the model’s ability to identify malicious activities in sequential data packets arriving in a specific order over time. This shows strong results across multiple datasets, achieving up to 99.98% accuracy on Kaggle EDGE_IIoT, 99.97% on Kaggle BoT-IoT and 98.4% on InSDN dataset with specific integrated GRU in PSO optimized GAN and IDS model demonstrating competitive performance compared to other models. The time complexity and scalability of existing models are constrained compared to the proposed model PSO-GRUGAN-IDS. Few existing approaches struggle with high computational demands and inefficiency as the network size or traffic volume grows. In contrast, the proposed model utilizes optimized algorithms with GRU GAN that minimize processing time and enhance scalability and enabling it to manage larger and more intricate datasets more effectively in real-time 5G SDN network environments, which is essential for high throughput, low latency and high-quality service in 5G SDN Networks.

## Limitations and potential solutions

### Addressing the risk of overfitting to synthetic data in PSO-GRUGAN-IDS

Risk of Overfitting: The PSO-GRUGAN-IDS model shows promising performance across various datasets, leveraging synthetic attack data generated by the GAN for training. However, a significant risk arises from potential overfitting to the synthetic distribution, leading to diminished generalization when faced with real-world, unseen data.

### Proposed solutions

#### Validation on unseen real-world data

- Diverse Dataset Utilization: Incorporate diverse and realistic datasets such as CICDDoS2019, KT-21, KD-P, and real-world traffic from different IoT and IIoT environments. This can help the model adapt to real-world variability and reduce dependency on synthetic data.
- Cross-Dataset Validation: Validate the model on multiple datasets beyond InSDN, ensuring robustness across different network environments and attack scenarios.

#### Data augmentation techniques

- Hybrid Data Mix: Use a balanced mix of real and synthetic data during training to avoid overfitting. This includes dynamically updating the training set with newly captured real-world traffic data.
- Domain Adaptation: Apply domain adaptation techniques to align the synthetic data distribution more closely with real-world data, enhancing the model’s adaptability to unseen attacks.

### Performance metrics validation

The performance metrics of the PSO-GRUGAN-IDS model, including high accuracy (up to 99.98% on Kaggle EDGE_IIoT, 99.97% on Kaggle BoT-IoT, 98.4% on InSDN dataset), highlight its potential. However, validating these results on unseen, real-world datasets can provide a more comprehensive evaluation of its true effectiveness. This approach will help assess the model’s generalization and fine-tune its parameters for optimal performance in real-time 5G SDN environments.

To mitigate the risk of overfitting to synthetic data, the PSO-GRUGAN-IDS framework should be rigorously validated on diverse real-world datasets. This ensures that the model remains effective and scalable in dynamic, real-time 5G network environments, fulfilling its primary objective of accurate and efficient intrusion detection, particularly for varying attack patterns and rapid changes in traffic. The overall performance analysis of this section shows that the PSO-GRUGAN-IDS model-based attack detection approach obtains better performance than other approaches for all the performance metrics.

## Conclusion

This research focuses on developing a robust IDS framework for 5G SDN to ensure secure and reliable communication. The unique attributes of 5G SDN, such as efficient resource utilization, rapid response times, and seamless data flow, introduce specific security challenges, including vulnerabilities that could jeopardize network availability and integrity. Overcoming these challenges requires an advanced system capable of effectively monitoring network traffic behavior. The primary aim of this study is to design an advanced deep learning-based Intrusion Detection System (IDS) to anticipate and identify emerging attacks within 5G SDN networks, facilitating real-time, continuous monitoring of network traffic. In pursuit of this objective, the research introduces the cutting-edge PSO-GRUGAN-IDS model, which utilizes the InSDN dataset to analyze and predict abnormal traffic patterns that could indicate malicious activity in SDN environments. A key focus is on optimizing the model’s performance by reducing false positives, detection time, and detection loss, thereby enhancing the accuracy of attack detection and maximizing throughput. Furthermore, the study emphasizes the importance of scalability, ensuring that the model can handle large volumes of data and adapt to the growing complexity of 5G SDN networks. This approach aims to not only improve security but also maintain network efficiency in dynamic, real-time 5G environments.

Performance analysis from the previous sections demonstrates that the PSO-GRUGAN-IDS model outperforms other methods across various metrics, achieving high accuracy (0.984) and throughput (2567 samples/second). Additionally, the model shows favorable results in terms of attack detection loss for GR and DR (0.69 and 0.66, respectively), RMSE (1.2), MAE (1.6), detection time (training in 185.1 s and detection in 2.464 s), and CPU utilization (4.449%). Moreover, the proposed PSO-GRUGAN-IDS model shows strong results across multiple datasets, achieving up to 99.98% accuracy on Kaggle EDGE_IIoT, 99.97% on Kaggle BoT-IoT, and 98.4% on InSDN dataset demonstrating competitive performance compared to other models. Performance analysis shows the model excels across various metrics, achieving high accuracy (up to 99.98%), low detection loss, and efficient processing times. The study recommends the PSO-GRUGAN-IDS for efficient monitoring of 5G SDN traffic, addressing various traffic patterns and ongoing changes in traffic, while ensuring effective intrusion detection.

## Future scope

- While the current study shows promising results, its performance could improve with more extensive datasets. Future research should focus on developing more efficient Lightweight Deep Learning models to manage and scale with growing volumes of attack data, ensuring robust detection capabilities in dynamic 5G SDN environments.
- Additionally, creating a lightweight security mechanism for integration into the three phases of 5G SDN network plans could help mitigate various attacks at different stages of the network.
- Expanding the study to monitor attack activities and vulnerabilities across multiple SDN controllers in 5G SDN networks is another potential area for future work. The present research is limited to a single SDN controller, but future efforts could extend to multi-controller SDN networks.
- Furthermore, continuous network behavior monitoring methodologies could be developed to provide real-time alerts to network developers, enabling timely updates to network security.

## Data Availability

http: //iotseclab.ucd.ie/datasets/SDN, https://www.kaggle.com/datasets/mohamedamineferrag/edgeiiotset-cyber-security-dataset-of-iot-iiot, https://www.kaggle.com/datasets/vigneshvenkateswaran/bot-iot.
